# Efficient deletion of multiple circle RNA loci by CRISPR‐Cas9 reveals *Os06circ02797* as a putative sponge for *OsMIR408* in rice

**DOI:** 10.1111/pbi.13544

**Published:** 2021-01-28

**Authors:** Jianping Zhou, Mingzhu Yuan, Yuxin Zhao, Quan Quan, Dong Yu, Han Yang, Xu Tang, Xuhui Xin, Guangze Cai, Qian Qian, Yiping Qi, Yong Zhang

**Affiliations:** ^1^ Department of Biotechnology School of Life Sciences and Technology Center for Informational Biology University of Electronic Science and Technology of China Chengdu China; ^2^ State Key Laboratory of Rice Biology China National Rice Research Institute Hangzhou China; ^3^ Sichuan Grass Industry Technology Research and Promotion Center Chengdu China; ^4^ School of Agricultural science Xichang University Xichang China; ^5^ Department of Plant Science and Landscape Architecture University of Maryland College Park MD USA; ^6^ Institute for Bioscience and Biotechnology Research University of Maryland Rockville MD USA

**Keywords:** CRISPR‐Cas9, large deletion, circle RNA, microRNA sponge, rice

## Abstract

CRISPR‐Cas9 is an emerging genome editing tool for reverse genetics in plants. However, its application for functional study of non‐coding RNAs in plants is still at its infancy. Despite being a major class of non‐coding RNAs, the biological roles of circle RNAs (circRNAs) remain largely unknown in plants. Previous plant circRNA studies have focused on identification and annotation of putative circRNAs, with their functions largely uninvestigated by genetic approaches. Here, we applied a multiplexed CRISPR‐Cas9 strategy to efficiently acquire individual null mutants for four circRNAs in rice. We showed each of these rice circRNA loci (*Os02circ25329*, *Os06circ02797*, *Os03circ00204* and *Os05circ02465*) can be deleted at 10% or higher efficiency in both protoplasts and stable transgenic T0 lines. Such high efficiency deletion enabled the generation of circRNA null allele plants without the CRISPR‐Cas9 transgene in the T1 generation. Characterization of the mutants reveals these circRNAs’ participation in salt stress response during seed germination and in particular the *Os05circ02465* null mutant showed high salt tolerance. Notably, the seedlings of the *Os06circ02797* mutant showed rapid growth phenotype after seed germination with the seedlings containing higher chlorophyll A/B content. Further molecular and computational analyses suggested a circRNA–miRNA–mRNA regulatory network where *Os06circ02797* functions to bind and sequester *OsMIR408*, an important and conserved microRNA in plants. This study not only presents genetic evidence for the first time in plants that certain circRNAs may serve as sponges to negatively regulate miRNAs, a phenomenon previously demonstrated in mammalian cells, but also provides important insights for improving agronomic traits through gene editing of circRNA loci in crops.

## Introduction

The development of genome editing tools such as ZFN, TALEN and CRISPR‐Cas systems has greatly boosted reverse genetics in plants (Ming *et al*., 2020; Ren *et al*., 2020; Schindele *et al*., 2020; Tang *et al*., 2017; Tang *et al*., 2019; Voytas, 2013; Yin *et al*., 2017; Zhang *et al*., 2019). While CRISPR‐Cas9 is widely used for knocking out protein‐coding genes, it has also enabled other genetic applications such as alteration of splicing sites (Li *et al*., 2019b; Xue *et al*., 2018), promoter editing for generating quantitative trait variation (Rodriguez‐Leal *et al*., 2017), editing upstream open reading frames (uORFs) for enhanced protein translation (Zhang *et al*., 2018) and creation of loss of function alleles of micro RNA (miRNA) genes (Bi *et al*., 2020; Zhou *et al*., 2017). CRISPR‐Cas technologies are especially powerful for dissecting the functions of non‐coding genes as null alleles can be generated through targeted deletions, which is difficult to achieve by conventional mutagenesis tools.

Circle RNAs (circRNAs) belong to a major class of non‐coding RNAs that have unique structure and functions in eukaryotes (Li *et al*., 2018). Unlike linear RNAs, circRNAs are single‐stranded circular RNAs that are mainly generated from a back‐splicing mechanism (Chen, 2020; Li *et al*., 2018). Interestingly, circRNAs can be derived from any genomic regions, including exonic, intronic and intergenic sequences (Chen, 2016). Due to lack of 3′ polyadenylated tails, circRNAs are largely absent from the classic RNA sequencing data sets. With advances of sequencing technologies in recent years, a great number of circRNAs have been discovered in different organisms. For example, over 180 000 circRNAs have been identified in human transcriptomes (Chen, 2020; Dong *et al*., 2018; Zheng *et al*., 2019). In plants, 38 938 and 40 311 circRNAs have been discovered in Arabidopsis and rice, respectively (Chu *et al*., 2018). The relatively smaller numbers of circRNAs reported in other plant species are probably due to limited investigation and incomplete genome annotation (Chu *et al*., 2018).

Genetic and genomic studies on circRNAs in animal and human systems have pointed to diverse functions, including transcription and splicing regulation, protein translation, and miRNA sponges (Chen, 2020; Li *et al*., 2018; Patop *et al*., 2019). An interesting function for circRNAs is to serve as miRNA sponges, where binding of miRNAs to circRNAs prevents miRNAs from targeting and suppressing their cognitive mRNAs (Salmena *et al*., 2011). Evidence supporting that circRNAs function as sponges for miRNAs was previously demonstrated in human cells (Huang *et al*., 2017; Li *et al*., 2019a; Memczak *et al*., 2013; Zheng *et al*., 2016a) and mouse cells (Hansen *et al*., 2013; Piwecka *et al*., 2017). These mammalian studies all pointed to complex and fascinating circRNA–miRNA–mRNA regulatory networks which may be widespread among eukaryotic organisms including plants.

In recent years, genome‐wide identification of circRNAs was reported in multiple plant species, such as *Arabidopsis* (Sun *et al*., 2016; Ye *et al*., 2015), rice (Lu *et al*., 2015; Ye *et al*., 2015; Ye *et al*., 2017), maize (Chen *et al*., 2018), tomato (Tan *et al*., 2017; Zuo *et al*., 2016), soybean (Zhao *et al*., 2017) and poplar (Liu *et al*., 2020). Computational tools such as PcircRNA_finder have greatly enabled the research on circRNA identification (Chen *et al*., 2016). Subsequently, multiple online databases were created to host the rapidly growing circRNA data in plants (Chu *et al*., 2018; Chu *et al*., 2017; Meng *et al*., 2019; Wang *et al*., 2019b; Ye *et al*., 2019). Despite the abundant putative circRNAs found in plants, to date only one circRNA has been functionally elucidated. In that study, a circRNA derived from *SEPALLATA3* was shown to regulate mRNA splicing through R‐loop formation and overexpression of this circRNA resulted in flowers with altered floral organ number in *Arabidopsis* (Conn *et al*., 2017).

Functional analysis of circRNAs in plants is at its infancy. Previous attempts to study circRNAs had relied on ectopic circRNA gene overexpression (Conn *et al*., 2017; Lu *et al*., 2015). It is thus of great urgency to develop a reliable reverse genetic approach for elucidating the function of circRNA genes in plants. In this study, we aimed to demonstrate a CRISPR‐Cas9‐based genetic knockout approach to study the function of circRNAs in rice. We reasoned that powerful phenotypic analysis and transcription profiling could be done using CRISPR‐Cas9‐generated circRNA mutants, which would help reveal the potential function of target circRNAs. Coupling targeted mutagenesis with transcription analysis, we sought to provide a first experimental evidence for circRNA–miRNA–mRNA regulatory networks in plants. Meanwhile, we also explored the editing of circRNA genes as a promising approach for improving agronomic traits in crops.

## Results

### Efficient deletions of rice circRNA loci with multiplexed CRISPR‐Cas9

**Figure 1:**
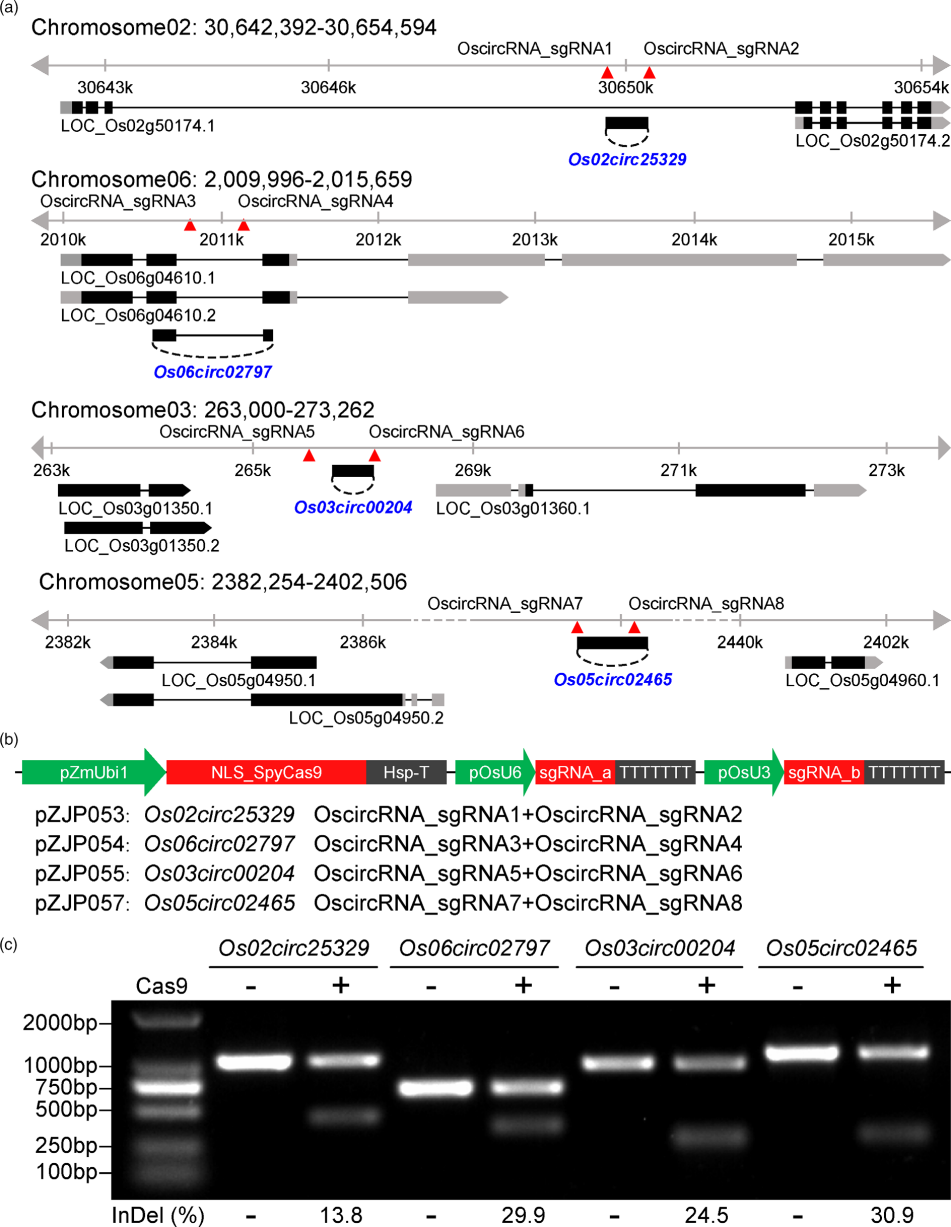
CRISPR‐Cas9‐mediated deletion of four circRNA loci in rice. (a) Genomic locations of four circRNA genes in this study. Exons are indicated as black boxes. UTRs are indicated as grey boxes. The sgRNA region of each circRNA is indicated by two red triangles. Note the circRNA genes (*Os02circ25329* and *Os06circ02797*) are located in intragenic introns, while the other two circRNA genes (*Os03circ00294* and *Os05circ02465*) are located in intergenic regions. (b) Schematics for four multiplexed CRISPR‐Cas9 T‐DNA vectors (pZJP053, pZJP054, pZJP055 and pZJP057) with each expressing two sgRNAs for targeted deletion of each circRNA gene. The Cas9 gene is expressed under a maize ubiquitin 1 promoter (pZmUbi1), and the sgRNAs are expressed under a rice U6 promoter (pU6) and U3 promoter (pU3), respectively. (c) Targeted chromosomal deletion of four circRNA loci in rice protoplasts. Compared to the untransformed protoplasts, chromosomal deletions of circRNA genes were detected by PCR in transformed protoplasts.

Based on a previous transcriptome‐wide analysis of circRNAs in rice (Lu *et al*., 2015), we chose four rice circRNA candidate loci for investigation in this study. These four circRNAs all have relatively high transcription abundance, indicating their importance in cellular function and possible roles as miRNA sponges. Two rice circRNA genes, *Os02circ25329* and *Os06circ02797*, are derived from introns of *Os02g50174* and *Os06g04610*, respectively (Figure 1a). The other two rice circRNA genes, *Os03circ00204* and *Os05circ02465*, are derived from intergenic regions: one (*Os03circ00204*) residing in between *Os03g01350* and *Os03g01360* and the other (*Os05circ02465*) residing in between *Os05g04950* and *Os5g04960* (Figure 1a). To generate null alleles of circRNA genes with minimal interference of the hosting genes or flanking genes, we designed a pair of single‐guide RNAs (sgRNAs) with targeting sites right at the edge or outside of the circRNA coding region for each circRNA gene (Figure 1a). Four multiplex T‐DNA vectors (pZJP053, pZJP054, pZJP055 and pZJP057) were generated for targeting these four circRNA genes of interest (Table 1, Figure 1b). In each construct, the Cas9 gene was expressed under a maize ubiquitin 1 promoter (pZmUbi1) and the two sgRNAs were each expressed under a rice U6 (pU6) and U3(pU3) promoters, respectively (Figure 1b). To assess editing activity of these vectors, we transiently transformed them into rice protoplasts and used polymerase chain reaction (PCR) to detect the products resulting from Cas9‐mediated chromosomal deletions at the circRNA loci. In each case, a smaller PCR band corresponding to the deletion product for each circRNA gene was detected, indicating that chromosomal deletions of circRNA genes occurred in all four cases (Figure 1c). Further quantification of the bands showed high‐frequency deletions, ranging from 13.8% to 30.9%, across these four circRNA loci (Figure 1c).

**Table 1:** Targeted rice circRNA information and deletion efficiency in T0 lines

circRNA ID	Genomic location	Construct	sgRNA ID	sgRNA sequence (PAM)	Tested T0 lines	Mutated T0 lines Total: number/ratio; Biallelic: number/ratio	T0 deletion lines number/ratio
Os02circ25329	Intron	pZJP053	OscircRNA_sgRNA1	gcagctctgacatgtgggcc**CGG**	43	25/58.1%; 20/46.5%	6/14.0%
			OscircRNA_sgRNA2	gtcccgcgcttcaaggaggt**AGG**		23/53.5%; 21/48.8%	
Os06circ02797	Exon–intron	pZJP054	OscircRNA_sgRNA3	gaactatccgaggagcagtac**TGG**	34	22/64.7%; 18/52.9%	3/8.8%
			OscircRNA_sgRNA4	gaatgcaacccctgcaaacat**TGG**		14/41.2%; 10/29.4%	
Os03circ00204	Intergenic	pZJP055	OscircRNA_sgRNA5	gcctatacccttgaagctggg**AGG**	46	23/50.0%; 17/37.0%	5/10.9%
			OscircRNA_sgRNA6	gcttgcgcacaatcttaacga**AGG**		27/58.7%; 24/52.2%	
Os05circ02465	Intergenic	pZJP057	OscircRNA_sgRNA7	gtggaaaagcagcatatgtgc**AGG**	44	31/70.5%; 26/59.1%	6/13.6%
			OscircRNA_sgRNA8	gactccattccattttgcag**TGG**		25/56.8%; 20/45.5%	

### High‐frequency generation of rice circRNA deletion mutants

We next conducted stable transgenesis of rice with the four multiplex CRISPR‐Cas9 constructs. For the pZJP053 construct targeting *Os02circ25329*, 43 independent T0 transgenic lines were analysed. Among them, 25 (58.1%) lines carried mutations at the left target site and 20 (46.5%) lines carried biallelic mutations at this site; 23 (53.5%) lines carried mutations at the right target site and 21 (48.8%) lines carried biallelic mutations at this site; 6 (14.0%) lines contained large chromosomal deletions due to the simultaneous cleavage of both sgRNAs (Table 1). For the pZJP054 construct targeting *Os06circ02797*, 34 independent T0 transgenic lines were analysed. Among them, 22 (64.7%) lines carried mutations at the left target site and 18 (52.9%) lines carried biallelic mutations at this site; 14 (41.2%) lines carried mutations at the right target sites and 10 (29.45%) lines carried biallelic mutations at this site; 3 (8.8%) lines contained large chromosomal deletion due to the simultaneous cleavage of both sgRNAs (Table 1). For the pZJP055 construct targeting *Os03circ00204,* 46 independent T0 transgenic lines were analysed. Among them, 23 (50.0%) lines carried mutations at the left target site and 17 (37.0%) lines carried biallelic mutations at this site; 27 (58.7%) lines carried mutations at the right target site and 24 (52.2%) lines carried biallelic mutations at this site; 5 (10.9%) lines contained large chromosomal deletions due to the simultaneous cleavage of both sgRNAs (Table 1). For the pZJP057 construct targeting *Os05circ02465*, 44 independent T0 transgenic lines were analysed. Among them, 31 (70.5%) lines carried mutations at the left target site and 26 (59.1%) lines carried biallelic mutations at this site; 25 (56.8%) lines carried mutations at the right target site and 20 (45.5%) lines carried biallelic mutations at this site; 6 (13.6%) lines contained large chromosomal deletions resulting from simultaneous cleavage of both sgRNAs (Table 1).

**Figure 2:**
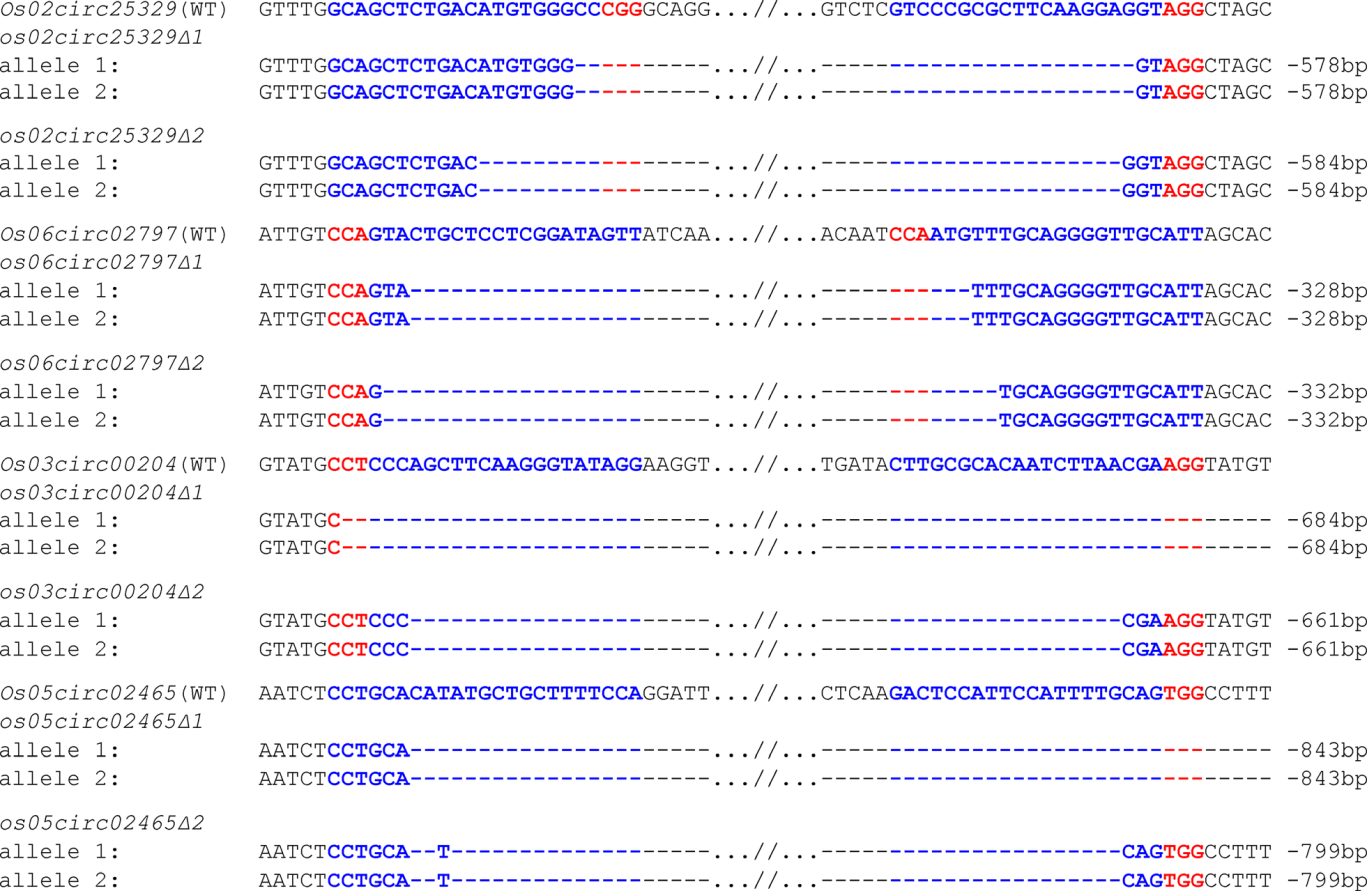
Genotypes of transgene‐free homozygous deletion lines in the T1 generation. Genotypes of homozygous lines containing chromosomal deletions of rice circRNAs were validated by PCR followed with Sanger sequencing. For each circRNA, two independent homozygous genotypes were identified. The CRISPR‐Cas9 transgenes were segregated out from these lines as confirmed by PCR.

Our analysis of T0 plants indicated that on average ~10% of T0 lines contained chromosomal deletions of circRNA genes, suggesting multiplex CRISPR‐Cas9 is a very efficient and reliable strategy to generate circRNA knockout mutants in rice. To obtain homozygous lines without the CRISPR‐Cas9 transgene, we followed the T0 deletion lines to the T1 generations. For each circRNA gene, we identified at least two independent T1 deletion lines (designated as ∆1 and ∆2) that carried different homozygous chromosomal deletion alleles with deletion sizes ranging from 328 bp to 843 bp, and these lines had segregated out the CRISPR‐Cas9 transgene (Figure 2). These transgene‐free T1 deletion lines were subsequently propagated for producing T2 seeds that would be used for all the following studies.

### Transcriptional analysis of circRNA genes and their parental or flanking genes in the rice deletion mutants

**Figure 3:**
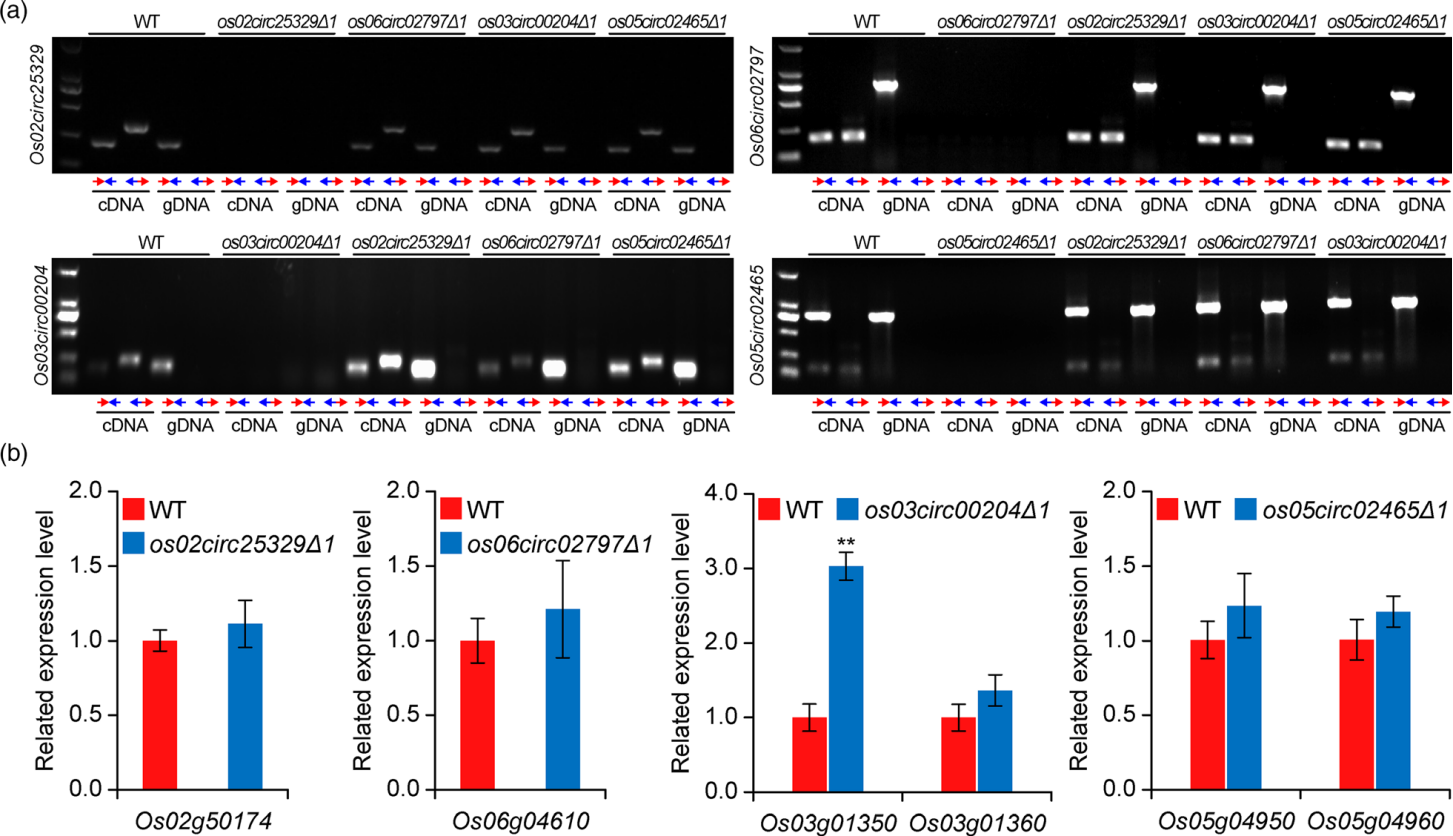
Transcriptional characterization of the circRNAs and their flanking genes in the rice circRNA mutants. (a) Detection of circRNAs in the wild‐type (WT) and circRNA mutants. Note two pairs of PCR primers were used for detecting each circRNA. The convergent PCR primers (indicated by two black face‐to‐face inward arrows) were used to detect genomic DNA (gDNA) and complementary DNA (cDNA) of each circRNA gene. The divergent PCR primers (indicated by two black back‐to‐back outward arrows) were used to specifically detect circRNAs. (b) (First) Relative gene expression of the *Os02circ25329*‐containing gene, *Os02g50174*, in the WT and circRNA mutant backgrounds. Relative gene expression of the *Os06circ02797*‐containing gene, *Os06g04610*, in the WT and circRNA mutant backgrounds. (Second) Relative expression of the two genomic genes (*Os03g01350* and *Os03g1360*) flanking the *Os03circ00204* gene in the WT and circRNA mutant backgrounds. (Third) Relative expression of the two genomic genes (*Os05g04950* and *Os05g04960*) flanking the *Os05circ02465* gene in the WT and circRNA mutant backgrounds. (Fourth) Gene expression was analysed by quantitative reverse transcription PCR (qRT‐PCR). The effort bars represent standard deviations of three biological replicates. Statistical significance is indicated by asterisks (** indicative of a *P*‐value < 0.01 by the student’s *t*‐test).

In our rice circRNA mutants, large chromosomal deletions should in principle eliminate the circRNA genes and their corresponding transcripts. To verify the absence of circRNA transcripts, we used the classic reverse transcription PCR (RT‐PCR) approach where two sets of PCR were used (Lu *et al*., 2015). The convergent PCR primers can amplify the complementary DNA (cDNA) as well as the genomic DNA (gDNA). The divergent PCR primers can amplify RNA in circular forms. Under both PCR settings, we were unable to amplify the target circRNA in its corresponding deletion mutant background (Figure 3a). As controls, anticipated PCR products for each circRNA were detected with convergent PCR primers from wild‐type (WT) cDNA and gDNA, as well as from the cDNA and gDNA from other mutant backgrounds, suggesting specific loss of circRNA transcripts in the corresponding mutant background (Figure 3a). Based on the data, we concluded that these circRNA deletion mutants are truly null alleles.

Due to their unique genomic locations and mode of production, circRNAs have inherent connection to their host genes or flanking genes. Hence, it is critical to know whether any of these host or flanking genes have altered expression in the circRNA mutants. For the two intronic circRNAs, *Os02circ25329* and *Os06circ02797,* quantitative RT‐PCR (qRT‐PCR) was used to assess the expression levels of the host genes *Os02g50174* and *Os06g04610*, respectively, in the WT and circRNA mutant backgrounds. No significant difference was observed in either case (Figure 3b), and the results were further confirmed by RNA sequencing (RNA‐seq) (Figure S1). For the two intergenic circRNAs, *Os03circ00204* and *Os05circ02465*, the two flanking genes at either side of each circRNA gene were investigated. Interestingly, the left flanking gene, *Os03g01350*, but not the right flanking gene (*Os03g01306*) was up‐regulated in the *os03circ00204∆1* mutant (Figure 3b). No significant difference was observed for the expression levels of both flanking genes, *Os05g04950* and *Os05g04960*, in the *os05circ02465∆1* mutant background (Figure 3b). These qRT‐PCR data were again corroborated by the independent RNA‐seq experiments (Figure S2). Our data suggest the expression of host or flanking genes was largely unaffected in the circRNA mutants, expect for the gene *Os03g01350* which flanks *Os03circ00204* in the upstream. As a result, caution must be taken when linking phenotype to genotype in the *os03circ00204∆1* mutant because the possible phenotype could be due to increased expression of *Os03g01350*, rather than loss of *Os03circ00204* function.

### Phenotypic analysis of circRNA mutants

**Figure 4:**
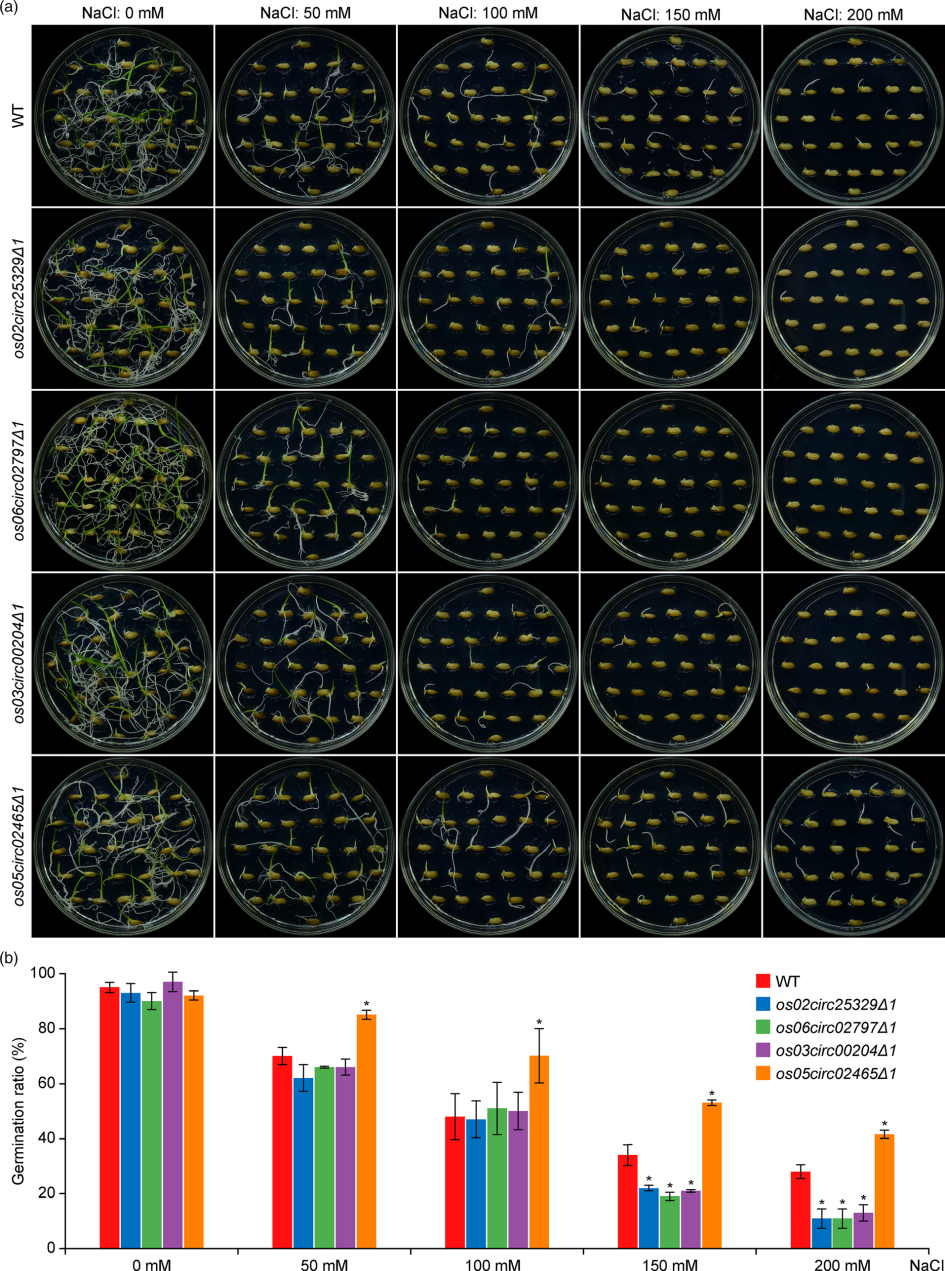
Differential responses of rice circRNA mutants to salt stress during seed germination. (a) A representative picture of seed germination under salt stress treatment for the WT plants and rice circRNA mutants. The seeds were germinated under different NaCl concentrations as indicated, and the picture was taken 7 days after sowing the seeds. (b) Quantification of germination rates for different genotypes under different NaCl concentrations. The error bars represent standard deviations (*n* = 3). Statistical significance is indicated by asterisks (* indicative of a *P*‐value < 0.05 by the Student *t*‐test).

Having confirmed circRNA mutants at both DNA and transcript levels, we sought to discover possible phenotypes related to the loss of function. The mutant plants of all four circRNAs were indistinguishable from the WT plants at maturation (Figure S3). Further characterization of the seeds did not reveal any significant difference in seed length, seed width and 1000‐grain weight between the mutants of all four circRNAs and the WT plants (Figure S4). We next looked for phenotypes on seed germination and early growth at the seedling stage. The mutants of circRNAs displayed comparable germinate rates (~90%) with that of the WT (Figure 4a and b). Treatment of salt stress, however, revealed divergent responses among circRNA mutants. For the mutants of *Os02circ25329*, *Os06circ02797* and *Os03circ00204*, they tolerated NaCl concentrations of 50 mm and 100 mm but became more sensitive to higher NaCl concentrations (150 mm and 200 mm) with significantly lower germination rates compared with the WT (Figure 4a and b). By contrast, the *Os05circ02465* mutants (*∆1* and *∆2*) showed clear tolerance to salt stress as it had higher germination rates than the WT at different NaCl concentrations (Figure 4a and b; Figure S5). In a similar experiment for assessing copper ion (Cu^2+^) stress in seed germination, no difference was found between the circRNA mutants and the WT plants (Figure S6). Together, these results supported specific and differential responses to salt stress in these circRNA mutants during seed germination. We also assessed the seedling growth under abiotic stress conditions. Neither salt stress nor heat stress resulted in detectable morphogenic phenotypes among circRNA mutants (Figures S7 and S8).

### RNA‐seq analysis of differentially expressed genes in rice circRNA mutants

**Figure 5:**
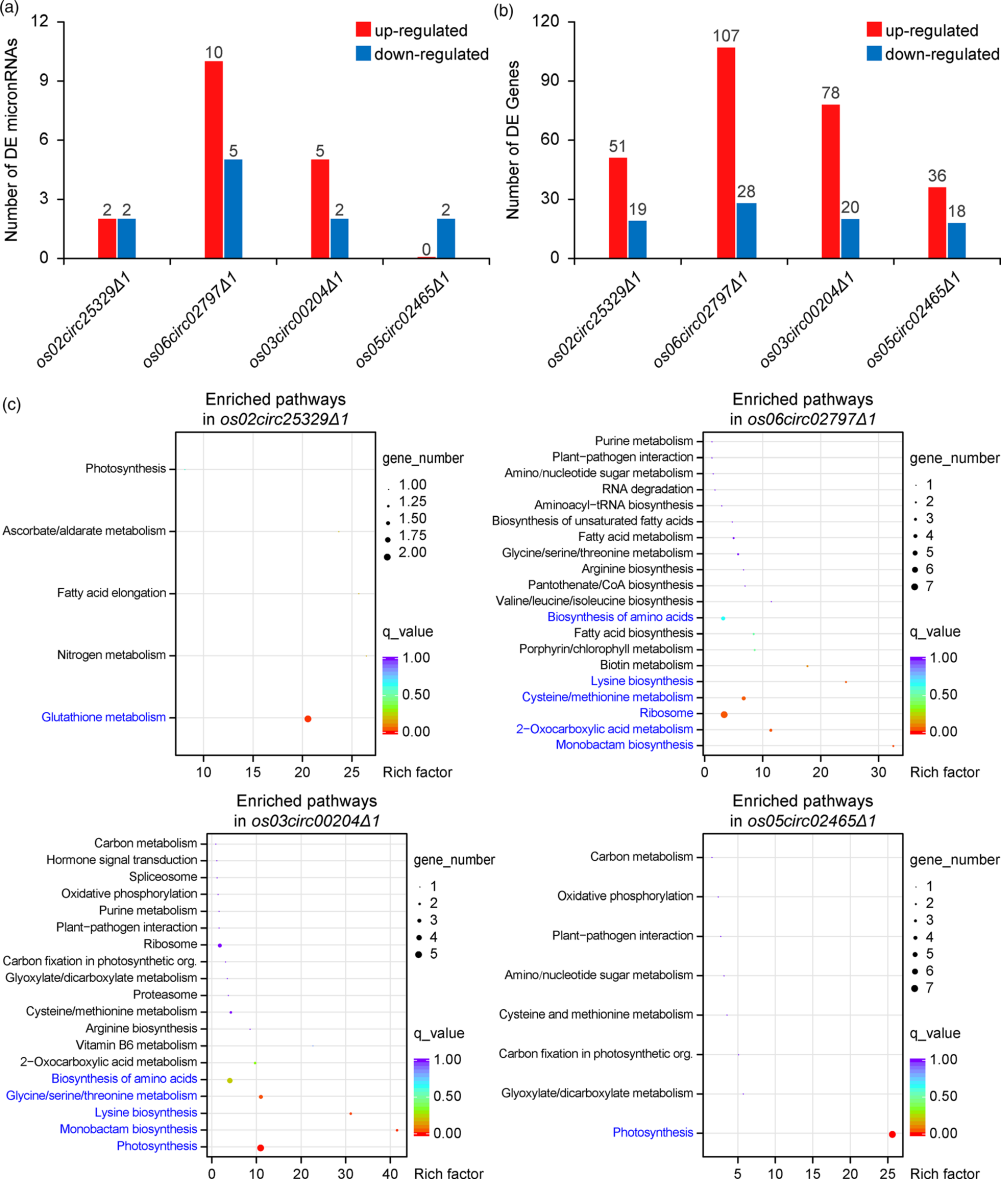
Transcriptome analysis of rice circRNA mutants. (a) Quantification for the numbers of differentially expressed miRNAs in the rice circRNA mutants as compared to the WT background. (b) Quantification for the numbers of differentially expressed protein‐encoding genes in the rice circRNA mutants as compared to the WT background. (c) Pathway enrichment analyses for the differentially expressed genes in four rice circRNA mutants.

The germination and seedling phenotype of circRNAs promoted us to investigate the underlining mechanisms for such phenotypes. We hypothesized that these circRNAs may regulate the abundance of miRNAs and protein‐coding mRNAs in rice. To this end, we conducted transcription profiling of small RNAs and protein‐coding mRNAs with corresponding small RNA‐seq and mRNA‐sq experiments. The small RNA‐seq experiment for small RNAs revealed differential expression of miRNAs. For example, 10 miRNAs were up‐regulated, and 5 miRNAs were down‐regulated in the *os06circ02797∆1* mutant, and it appeared more miRNAs were differentially expressed in the *os06circ02797∆1* mutant than in other circRNA mutants (Figure 5a, Table S1). The mRNA‐seq experiment for mRNAs showed a similar trend where the *os06circ02797∆1* mutant had the most differentially expressed mRNAs, with 107 mRNAs up‐regulated and 28 mRNA down‐regulated (Figure 5b, Table S2). Further analysis of both RNA‐seq data indicated that the differential miRNA expression was correlated with the expression of potential target genes in the circRNA mutants (Tables [Link], [Link], [Link], [Link], [Link]).

We further did heatmap and pathway enrichment analyses individually for differentially expressed genes in all four circRNA mutants. The trends of gene up‐regulation and down‐regulation were generally reproducible among three biological replicates (Figures S9, S10, S11 and S12). In the *os02circ025329∆1* mutant, the differentially expressed genes (Figure 5b, Table S2) were most enriched for the Glutathione metabolism pathway (Figure 5c, Table S7). In the *os06circ02797∆1* mutant, the differentially expressed genes (Figure 5b, Table S2) were mostly enriched for ribosome (7 chloroplast ribosomal proteins) and amino acid metabolism pathways (Figure 5c, Table S7). In the *os03circ00204∆1* mutant, the differentially expressed genes (Figure 5b, Table S2) were mostly enriched for the photosynthesis and amino acid metabolism pathways (Figure 5c, Table S7). In the *os05circ02465∆1* mutant, the differentially expressed genes (Figure 5b, Table S2) were mostly enriched for the photosynthesis pathway (Figure 5c, Table S7). These transcription profiling analyses suggest that these circRNAs are likely to participate in different cellular metabolism and signalling pathways independent from each other.

### Os06circ02797 negatively regulates OsMIR408 expression

**Figure 6:**
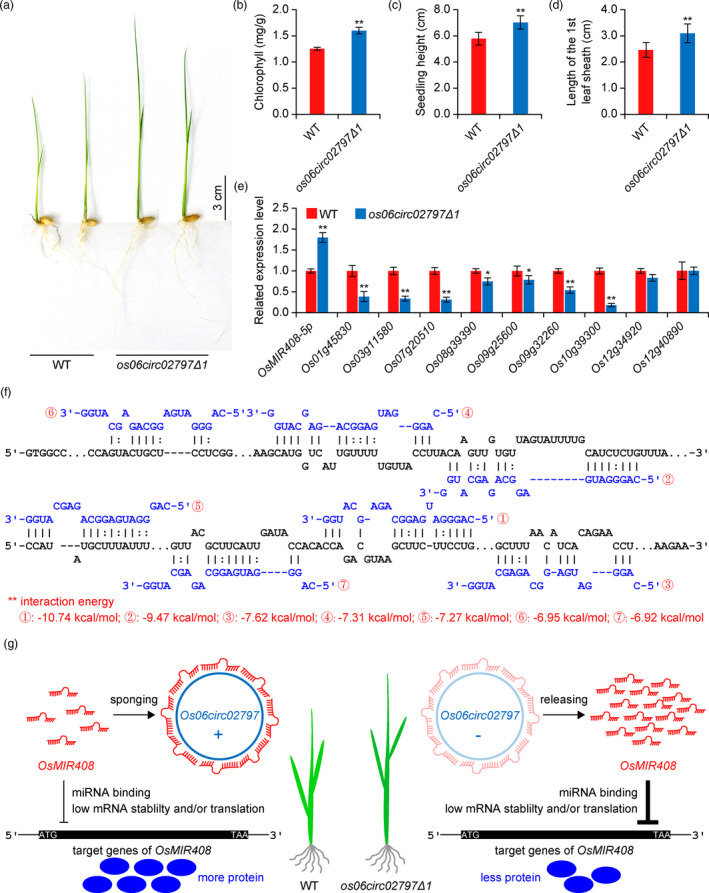
*Os06cic02797* negatively regulates *OsMIR408* expression. (a) Phenotypes of 7‐day‐old WT plants and *os06circ02797∆1* mutants. (b). Quantification of chlorophyll A/B content in the WT plants and *os06circ02797∆1* mutants. Error bars represent standard deviations (*n* = 3). (c) Quantification for the height of seedlings in the 7‐day‐old WT plants and *os06circ02797∆1* mutants. Error bars represent standard deviations (*n* = 30). (d) Quantification for the length of 1^st^ leaf sheath in the 7‐day‐old WT plants and *os06circ02797∆1* mutants. Error bars represent standard deviations (*n* = 30). (e) Relative expression of miR408‐5p and 9 potential targeting genes in the WT and *os06circ02797∆1* mutant backgrounds. Error bars represent standard deviations (*n* = 3). (f) Bioinformatic analysis of seven *OsMIR408*‐binding sites in the *Os0circ02797* circRNA using a web tool (http://www.rna‐society.org/raid/; The parameters are set as follows: Number of (sub)optimal interactions: 10, Suboptimal interaction overlap: can overlap in query, and Others: default). (g) A model depicting the *Os06circ02797* circRNA functions as a sponge for *OsMIR408* to negatively regulate its function in rice. Statistical significance is indicated by asterisks (* indicative of a *P*‐value < 0.05 by Student’s *t*‐test; ** indicative of a *P*‐value < 0.01 by Student’s *t*‐test).

At the seedling stage of phenotypic analysis, we found the seedlings of *os06circ02797∆1* were greener and taller than the WT seedlings (Figure 6a), indicating fast post‐germination growth. By quantification of the chlorophyll contents, we indeed found the *os06circ02797∆1* plant had higher concentration of chlorophyll A and B content than the WT plants (Figure 6b; Figure S13). The overall length of 1st leaf sheath of the mutant seedlings significantly surpassed those of the WT seedlings (Figure 6c and d). Moreover, the *os06circ02797∆1* plants were consistently taller than the WT plants under the salt stress and heat stress (Figures S7 and S8).

Meanwhile, we were curious about the possible mechanisms for these circRNAs to regulate a large set of differentially expressed genes, either directly or indirectly, as revealed by the small RNA‐seq and mRNA‐seq experiments. Given the *os06circ02797∆1* mutant showed more differentially expressed miRNAs and mRNAs and the mutant showed phenotypes in both seed germination and seedling growth, we decided to focus on *Os06circ02797* in the subsequent analysis. Equipped with RNA‐seq data for both miRNAs and mRNAs in this mutant background, we sought to explore the putative circRNA–miRNA–mRNA regulatory networks concerning *Os06circ02797*. Interestingly, overexpression of miR408 in *Arabidopsis* resulted in elevated levels of chlorophyll A and B and as well as elongated the hypocotyl (Zhang *et al*., 2011), a similar phenotype found in our *os06circ02797∆1* rice mutant (Figure 6b). These results suggest that *Os06circ02797* may function as sponges to sequester *OsMIR408* and hence negatively regulate *OsMIR408*. To test this hypothesis, we did qRT‐PCR to quantify the relative expression of *OsMIR408* in both the *os06circ02797∆1* mutant and the WT plants. Indeed, we observed higher expression of *OsMIR408* in the *os06circ02797∆1* mutant (Figure 6e). Furthermore, qRT‐PCR analyses showed that seven of nine putative *OsMIR408* target genes had markedly reduced expression in the *os06circ02797∆1* mutant (Figure 6e). These trends of altered gene expression were further validated by the mRNA‐seq data (Figure S14, Table S4). Remarkably, a bioinformatic analysis revealed that *Os06circ02797* has many putative binding sites for *OsMIR408* (Figure 6f). Altogether, these data strongly support an *Os06circ02797*‐*OsMIR408*‐mRNA regulatory network in rice, in which *Os06circ02797* functions as a sponge for *OsMIR408*, which probably helps fine‐tune the expression of *OsMIR408* target genes (Figure 6g).

## Discussion

CRISPR‐Cas genome editing systems are becoming standard molecular tools for reverse genetics in plants. Targeted small insertions and deletions (indels) by a single‐guide RNA are often sufficient for destroying the function of a protein‐coding gene due to the capability of introducing frameshifts and early stop codons (Ren *et al*., 2019; Tang *et al*., 2018; Tang *et al*., 2019; Zhang *et al*., 2019; Zhong *et al*., 2020). However, for non‐coding RNAs such as miRNAs, long non‐coding RNAs (lncRNAs) and circRNAs, introducing small indels may not yield loss of function. Previously, we showed that CRISPR‐Cas9‐mediated 1 bp indels at miRNA genes contributed to the generation of mutated miRNAs that may retain their original function (Zhou *et al*., 2017). Hence, in this study we applied a multiplexed editing strategy to deletion the entire coding regions of circRNAs in rice. We achieved high deletion frequency (over 10%) of all four circRNA loci in both protoplasts and stable T0 lines. This facilitated the generation of transgene‐free homozygous circRNA deletion mutants for functional investigation. Due to circRNAs’ unique nature of genesis, it is critical to make sure that their hosting or flanking genes are not affected by the chromosomal deletions. As our data showed, the expression of these genes in the circRNA knockout mutants is largely unaffected. Therefore, our study provided a straightforward reverse genetics pipeline for studying the function of circRNAs in plants.

Given so little was known for circRNA function in plants, we focused our phenotypic analysis on seed germination and seedling growth in the circRNA mutants with and without abiotic stresses. While knocking out these four circRNAs did not change seed germination rates under the normal condition, we found differential responses to salt stress during seed germination of the mutants. The mutants of three circRNAs, *Os02circ25329*, *Os06circ02797* and *Os03circ00204* showed high sensitivity to high concentration of NaCl (150 mm and 200 mm) (Figure 4). By contrast, the mutant of *Os05circ02465* showed high tolerance to salt stress when compared to the WT (Figure 4). It is rather surprising that mutants of all four circRNAs displayed seed germination phenotypes under salt stress. Interestingly, for these circRNA mutants, we did not find altered germination rates under Cu^2+^ stress conditions, nor did we find altered growth of the seedlings under salt stress. These data collectively suggest specific roles of these circRNAs in salt stress responses during seed germination. It, however, requires further studies to reveal the specific mechanisms that underline these circRNAs’ involvement in seed germination under salt stress.

Seed germination is a complex process that involves breaking dormancy, water imbibition, breakdown of storage proteins, metabolite and nutrient mobilization, etc (Rajjou *et al*., 2012; Ravindran and Kumar, 2019). While we did not conduct molecular analysis (e.g. transcriptional profiling) during seed germination in the circRNA mutants, we hypothesized that these circRNAs would most likely function through regulating miRNAs and mRNAs, and such regulatory modes could be investigated from tissues at later growth stages. Thus, we used mature leaf tissues of the circRNA mutants and WT plants for conducting the small RNA‐seq and mRNA‐seq experiments. Indeed, we have identified many miRNAs and mRNAs that are differentially expressed among different circRNA mutants. These data support our hypothesis for circRNAs’ regulatory roles in gene expression. However, further investigation is needed for validating and understanding such regulatory roles. It is possible that the significance of circRNAs’ regulatory roles may be masked in rice mature tissues since the mutants at this stage are morphologically indistinguishable from the WT plants (Figure S3). In the future, it is worth conducting RNA‐seq experiments using these circRNA mutants during seed germination under salt stress, which may help explain the different levels of sensitivity of circRNA mutants to salt stress.

Among all four circRNAs, only mutants of *Oscirc02797* showed morphological phenotypes at the seedling stage. The mutants grow faster than the WT plants and are greener than the WT plants due to higher chlorophyll content (Figure 6a‐d). The increased height and chlorophyll content phenotypes in the seedlings of *oscirc02797∆1* mutant in rice were similar to the miR408 overexpression phenotype in *Arabidopsis* (Song *et al*., 2017; Zhang *et al*., 2011). Since miR408 is very conserved among higher plants (Axtell and Bowman, 2008; Kozomara and Griffiths‐Jones, 2011; Zhang *et al*., 2017), we reasoned the observed phenotype in rice mutants might be due to *Oscirc02797*’s negative regulation of *OsMIR408*. Indeed, we found *OsMIR408* was up‐regulated and many of its putative mRNA targets were down‐regulated in the *oscirc02797∆1* mutant (Figure 6e and Figure S14). Furthermore, computational analysis revealed there are at least seven putative *OsMIR408*‐binding sites on *Oscirc02797*, providing strong evidence supporting that *Oscirc02797*’s function as sponges for *OsMIR408* (Figure 6g; Table S8). Under this model, *Oscirc02797* functions to sequester *OsMIR408* and prevent it from targeting its mRNA targets. In the *oscirc02797∆1* mutant, such sequestering effects are removed, resulting in more *OsMIR408* molecules available to target its mRNA targets and down‐regulate their expression (Figure 6g). This sponger model is supported by multiple lines of evidence including the gene expression relationship revealed by qRT‐PCR and RNA‐seq, and multiple putative binding sites of *OsMIR408* on *Oscirc02797* revealed by computational analysis, and phenotypes of *Oscirc02797* mutants. Previously, evidence supporting circRNAs’ role as sponges for miRNAs was only provided in mammalian systems (Hansen *et al*., 2013; Huang *et al*., 2017; Li *et al*., 2019a; Memczak *et al*., 2013; Piwecka *et al*., 2017; Zheng *et al*., 2016a). Here, we provide a first genetic evidence for a similar phenomenon in plants. In the future, it will be interesting to investigate whether similar regulatory networks mirroring *Oscirc02797*‐*OsMIR408*‐mRNA exist in other plant species. Given *OsMIR408* has many putative target genes, it is also possible for multiple circRNAs to regulate *OsMIR408* as spongers. Hence, it is worthwhile to identify additional circRNAs that function as molecular sponges for *OsMIR408* and other miRNAs to in rice. Furthermore, it will be important to discover similar and additional circRNA–miRNA–mRNA regulatory networks in other plant species.

Interestingly, our small RNA‐seq and mRNA‐seq experiments showed that the circRNA mutants only affected a small subset of miRNAs and mRNAs (Figure 5a and b), when compared to miRNA mutants that we studied previously (Zhou *et al*., 2017). In our previous rice miRNA knockout study, we found that CRISPR‐Cas9‐mediated deletion of miRNA genes often resulted in perturbation of many other miRNAs, suggesting an interconnection in miRNA biogenesis (Zhou *et al*., 2017). While this phenomenon warrants more investigation, it shows the potential challenges of using miRNA editing in crop breeding, due to the pleiotropy observed at the molecular level. Given knocking out of a circRNA seems to change a small number of genes, it is appealing to use circRNA knockout to fine‐tune the expression of these genes in crop breeding. Our study indeed provides some insights towards this. For example, knocking out *Os05circ02465* resulted in high tolerance to salt stress during seed germination, and importantly, such an agronomic trait could be directly introduced into an elite rice cultivar by gene editing. With climate changes and frequent seawater intrusion into farmlands, this could be a really valuable trait, especially when the seeds are directly sown into the soil. In a second example, knocking out *Oscirc02797* promoted rapid seeding growth after seed germination, which is potentially another beneficial agronomic trait for use in elite rice cultivars. Together, our study provided two circRNA genes as potential trait genes for future rice improvement.

## Conclusion

In this study, we demonstrated CRISPR‐Cas9‐mediated chromosomal deletion as an effective means to generate knockout mutants of circRNAs in rice. To our knowledge, this is the first report of circRNA gene editing in plants. Notably, our research revealed an *Oscirc02797*‐*OsMIR408*‐mRNA regulatory network involved in seedling development in rice. This study thus provided a first genetic evidence for a plant circRNA to function as molecular sponges to sequester a miRNA. We also showed that knocking out *Os05circ02465* resulted in high salt tolerance and knocking out *Oscirc02797* resulted in fast seeding growth, providing two rice endogenous circRNAs as potential trait targets for gene editing‐based rice molecular breeding. Together, we demonstrated a practical gene editing method for reverse genetic study of plant circRNAs, revealed a putative circRNA–miRNA–mRNA regulatory network in rice, and shed light on crop improvement by editing circRNA genes in plants.

## Experimental procedures

### Plant materials

The rice cultivar Nipponbare (*Oryza sativa L. japonica*) was used as the WT control and transformation host. The T2 generation of homozygote mutants without transgene was used for phenotypic and molecular characterization.

## Construction of the vectors

We used Cas9 expression backbone vector pZHY988 (p35S::Cas9::Hsp T‐pOsU6::sgRNA::pT) (Tang *et al*., 2016; Wang *et al*., 2019a) for this study. To make a dual‐sgRNAs vector, two expression cassettes of OsU6 promoter‐gRNA scaffold‐OsU6 terminator were cloned into pZHY988 using fusion PCR followed by ligation. All the primers are listed in Table S9. Taking the vector pZJP053 as an example, the DNA fragment was obtained from PCR products using primers Os02circ25329‐sgRNA‐P1F and Os02circ25329‐sgRNA‐P2R using template pZJP046 (Zhou *et al*., 2019). The PCR fragment was cut by Bsa I and then cloned into Bsa I‐digested pZHY988. T‐DNA vectors pZJP054, pZJP055 and pZJP057 were made in a similar fashion.

### Rice protoplast transformation and stable transformation

Rice protoplast transformation was performed as described previously (Tang *et al*., 2016; Zhang *et al*., 2013; Zhong *et al*., 2018). After transformation, rice protoplasts were incubated at 28 °C for 2 days before DNA extraction. The T‐DNA constructs were introduced into *Agrobacterium* EHA105 by the freeze‐thaw method. Rice stable transformation was carried out as according to a previously published protocol (Tang *et al*., 2016; Zhou *et al*., 2019).

### Detection of targeted gene mutations

Genomic DNA was extracted from protoplasts or transgenic plants by using the CTAB method (Murray and Thompson, 1980; Zheng *et al*., 2016b). Genomic regions of targeted sites were amplified with specific primers for detection of chromosomal deletions (Table S9) (Zhong *et al*., 2020). The PCR products were analysed on 1% agarose gels. T0 and T1 mutant lines were further genotyped by Sanger sequencing.

### Small RNA sequencing and mRNA transcriptome sequencing

The mutants and WT plants were chosen for small RNA sequencing and mRNA transcriptome sequencing (Wang *et al*., 2019a; Zhou *et al*., 2017). Whole plants of 40‐day‐old grown in the growth chamber under long‐day conditions (16‐h light at 28 °C and 8‐h dark at 22 °C) were collected. Three independent plants for each mutant were chosen for library construction, sequencing and analysis. Small RNA and mRNA transcriptome sequencing were done using Illumina HiSeq 2500 platform at Biomarker Technologies Co. Ltd. Data processing and analysis were carried out with the BMKCloud service (http://www.biocloud.net/).

### RNA extraction and qRT‐PCR

Total RNA was extracted using TRIzol Universal Reagent (Tiangen, China), treated with DNase I and then used for cDNA synthesis. For miRNA detection, miRNA cDNA synthesis was carried out using the miRcute miRNA cDNA kit (Tiangen, China) per the manufacturer’s instructions. SYBR green‐based qRT‐PCR was performed using a specific forward primer (Table S9) and a universal reverse primer provided by the kit (Tiangen, China). For qRT‐PCR of mRNA, reverse transcription (RT) was carried out using HiScript III RT SuperMix for qPCR (Vazyme, China), and qRT‐PCR was performed with ChamQ Universal SYBR qPCR Master Mix (Vazyme, China) according to the manufacturer’s instructions. Actin mRNA was used as an internal control. The relative levels of gene expression were calculated using the 2^−△△Ct^ method. Three biological replicates (three independent mutant T2 seedlings) were examined to ensure reproducibility. The experiments were performed 3 times independently with similar results.

### Chlorophyll content analysis

Mature seeds of Os06circ02797 mutant and WT were geminated for 2 days at 37 °C in the dark, and the germinated seeds were planted into containers in the growth chamber under a long‐day condition (16‐h light at 28 °C and 8‐h dark at 22 °C). After 7 days, the seedlings were harvested for further analysis. Chlorophyll was extracted and measured according to a previously described protocol (Wei *et al*., 2017). Briefly, seedling tissue was homogenized in 80% acetone at 4 °C, the homogenates centrifuged, and fluorescence measured at 662, 645 and 440 nm with Fluorescence Spectrometer HITACHI U2910 (JAPAN).

### Seed germination analysis under Na^+^/Cu^2+^ stress

The seeds of circRNA mutants and WT were put on the culture dishes and germinated under different NaCl/CuSO_4_ concentrations. For each treatment, 30 seeds per dish with three replicates were used under a long‐day condition (16‐h light at 28 °C and 8‐h dark) at 22 °C. Germination rates were counted, and photographs were taken at 7 days after sowing the seeds.

### Analysis of seedling response to stress

Seeds were geminated for 2 days at 37 °C in the dark. Germinated seeds were then transferred to culture plates, and seedlings were grown under the growth chamber under a long‐day condition (16‐h light at 28 °C and 8‐h dark at 22 °C). For NaCl stress, the seedlings were treated with different NaCl concentrations on the 9th day, with photographs taken at 7 days after NaCl treatment. For high‐temperature stress, the seedlings were transferred to a high‐temperature growth chamber (42 °C), with photographs taken at 7 days after 42 °C treatment.

## Conflicts of interest

The authors declare no competing interests.

## Authors’ contributions

Y.Z. proposed the project and designed the experiments. J.Z., M.Y., X.T. and X.X. designed sgRNAs and constructed all the vectors. M.Y., Y.Z., Quan Q., D.Y., H.Y. and X.X. did the rice stable transformation. J.Z., M.Y., D.Y., G.C. and Qian Q. did the rice mutants identification and analysis. J.Z. and M.Y. performed RNA‐seq experiments. Y.Z., Y.Q., J.Z. and Qian Q. analysed the data. Y.Z., Y.Q. and J.Z. wrote the manuscript. All authors participated in discussion and revision of the manuscript. All authors read and approved the final manuscript.

## Supporting information


**Figure S1** mRNA‐Seq based quantification of the expression levels of parental genes in rice circRNAs mutants.
**Figure S2** mRNA‐Seq based quantification of the expression levels of flanking genes in rice circRNAs mutants.
**Figure S3** Phenotype of mature rice circRNAs mutants.
**Figure S4** Phenotype of seeds in rice circRNAs mutants.
**Figure S5** Responses of *os05circ02465∆2* mutants to salt stress during seed germination.
**Figure S6** Germination response to CuSO_4_ stress in rice circRNAs mutants.
**Figure S7** Seedling growth of rice circRNAs mutants to salt stress.
**Figure S8** Seedling responses to high temperature in rice circRNAs mutants.
**Figure S9** Heat map clustering of differentially expressed genes in *os02circ25329∆1* mutant.
**Figure S10** Heat map clustering of differentially expressed genes in *os06circ02797∆1* mutant.
**Figure S11** Heat map clustering of differentially expressed genes in *os03circ00204∆1* mutant.
**Figure S12** Heat map clustering of differentially expressed genes in *os05circ02465∆1* mutant.
**Figure S13** Phenotypes of 7‐day old WT, *os06circ02797∆1* and *os06circ02797∆2* plants.
**Figure S14** The expression levels of *OsMIR408* and its putative target genes in *os06circ02797∆1* mutant.

**Table S1** The differential expression of miRNAs in rice circRNA mutants via small RNA‐Seq.

**Table S2** The differential expression of mRNA in rice circRNA mutants via mRNA‐Seq.

**Table S3** The differential expression miRNAs and their potential targets in *os02circ25329∆1* mutants.

**Table S4** The differential expression miRNAs and their potential targets in *os06circ02797∆1* mutants.

**Table S5** The differential expression miRNAs and their potential targets in *os03circ00204∆1* mutants.

**Table S6** The differential expression miRNAs and their potential targets in *os05circ02465∆1* mutants.

**Table S7** KEGG enrichment for the differentially expressed genes in four rice circRNA mutants.

**Table S8** Bioinformatics analysis rice miRNAs potentially targeted by Os06circ02797.

**Table S9** Oligos used in this study.

## Data Availability

The raw data of deep sequencing have been deposited to the Sequence Read Archive in National Center for Biotechnology Information (NCBI) under the accession numbers PRJNA655812 and PRJNA655827.
