# Supplementary material for: Efficient deletion of multiple circle RNA loci by CRISPR‐Cas9 reveals Os06circ02797 as a putative sponge for OsMIR408 in rice
Source: Plant Biotechnol J. 2021 Jan 28;19(6):1240–52. doi: 10.1111/pbi.13544 (PMC8196656; doi:10.1111/pbi.13544)
Supplement: Supplementary file 1 — Figure S1 mRNA‐Seq based quantification of the expression levels of parental genes in rice circRNAs mutants. Figure S2 mRNA‐Seq based quantification of the expression levels of flanking genes in rice circRNAs mutants. Figure S3 Phenotype of mature rice circRNAs mutants. Figure S4 Phenotype of seeds in rice circRNAs mutants. Figure S5 Responses of os05circ02465∆2 mutants to salt stress during seed germination. Figure S6 Germination response to CuSO4 stress in rice circRNAs mutants. Figure S7 Seedling growth of rice circRNAs mutants to salt stress. Figure S8 Seedling responses to high temperature in rice circRNAs mutants. Figure S9 Heat map clustering of differentially expressed genes in os02circ25329∆1 mutant. Figure S10 Heat map clustering of differentially expressed genes in os06circ02797∆1 mutant. Figure S11 Heat map clustering of differentially expressed genes in os03circ00204∆1 mutant. Figure S12 Heat map clustering of differentially expressed genes in os05circ02465∆1 mutant. Figure S13 Phenotypes of 7‐day old WT, os06circ02797∆1 and os06circ02797∆2 plants. Figure S14 The expression levels of OsMIR408 and its putative target genes in os06circ02797∆1 mutant. [file PBI-19-1240-s005.pptx]

## Slide 1
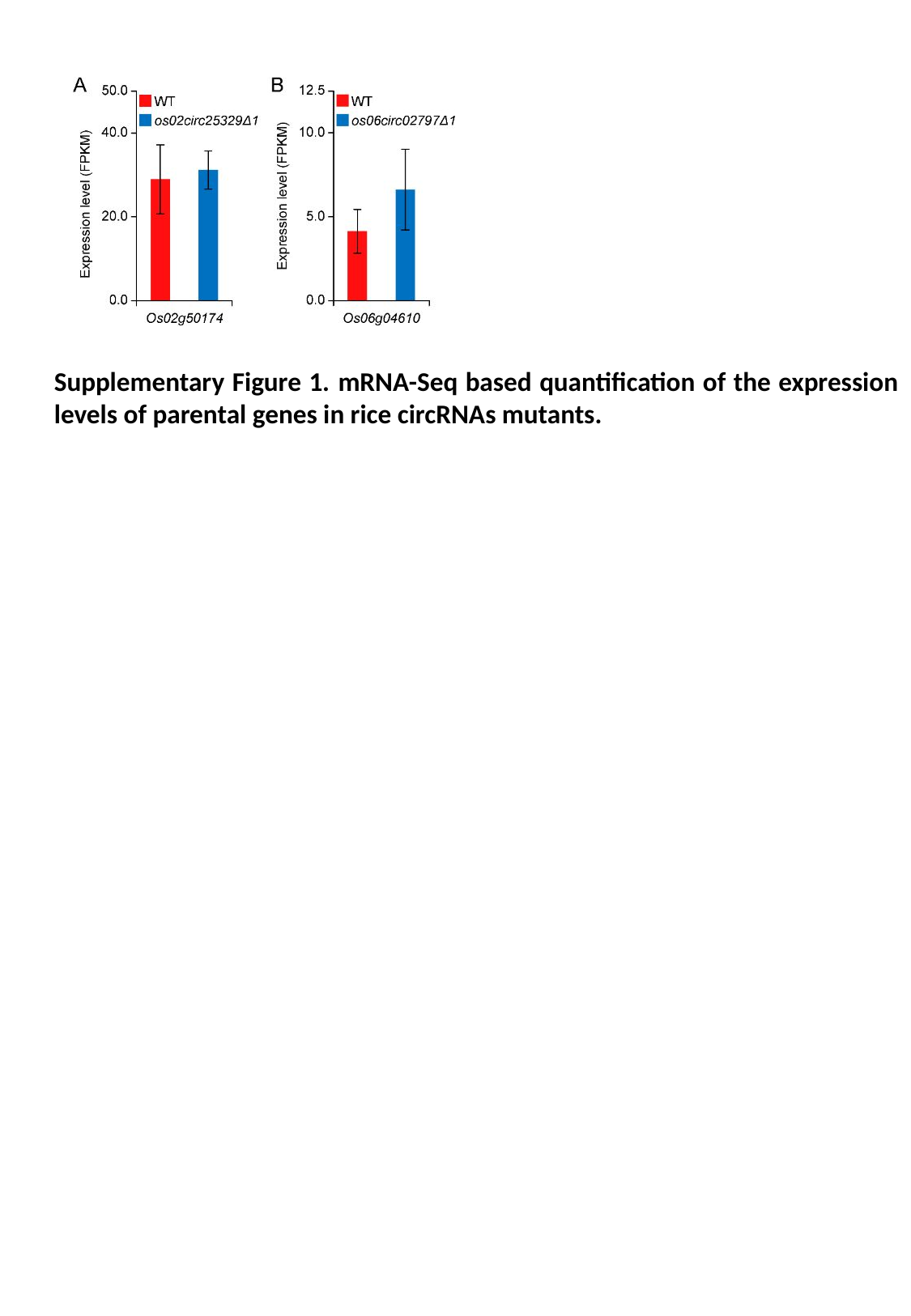

Supplementary Figure 1. mRNA-Seq based quantification of the expression levels of parental genes in rice circRNAs mutants.

## Slide 2
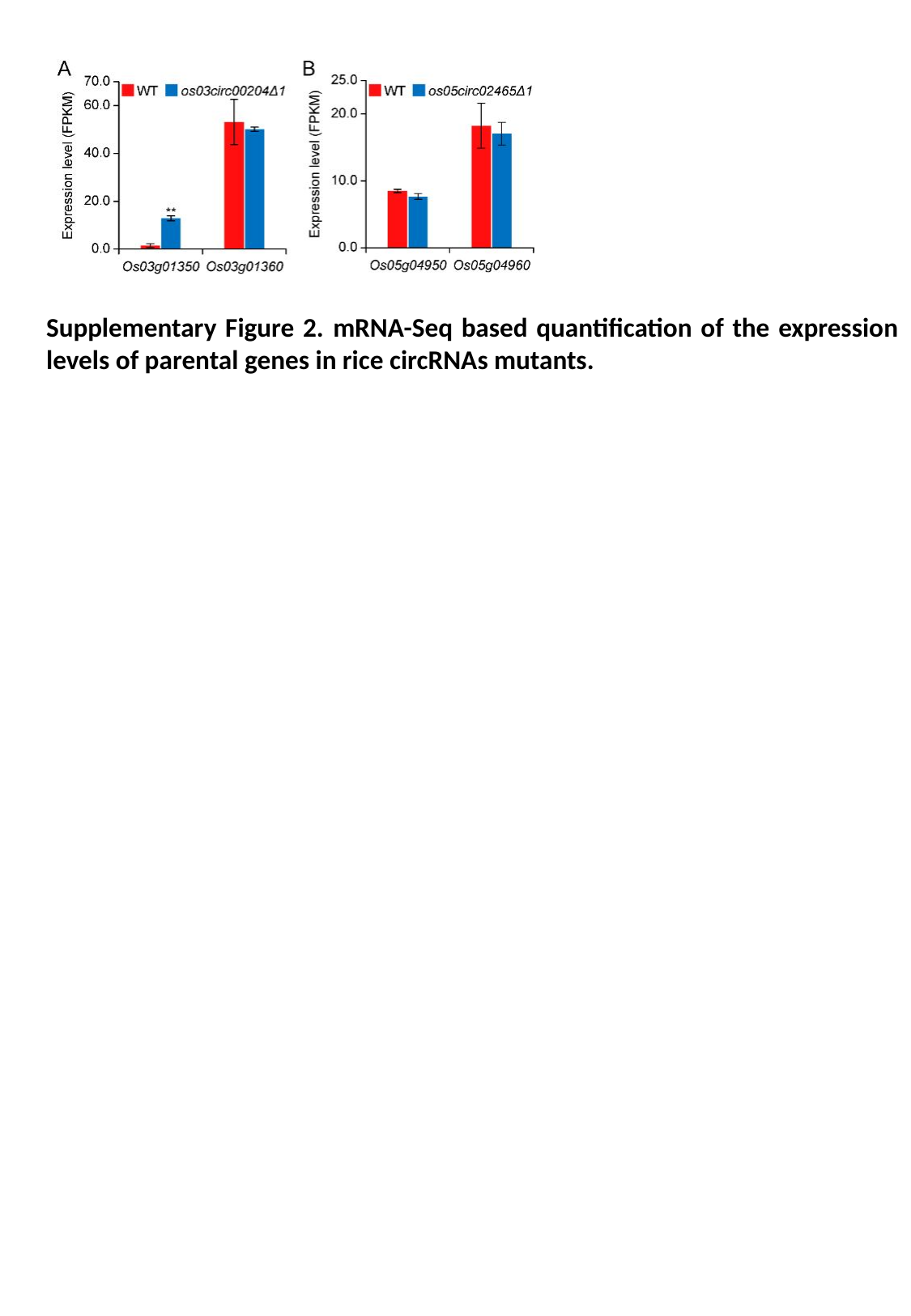

Supplementary Figure 2. mRNA-Seq based quantification of the expression levels of parental genes in rice circRNAs mutants.

## Slide 3
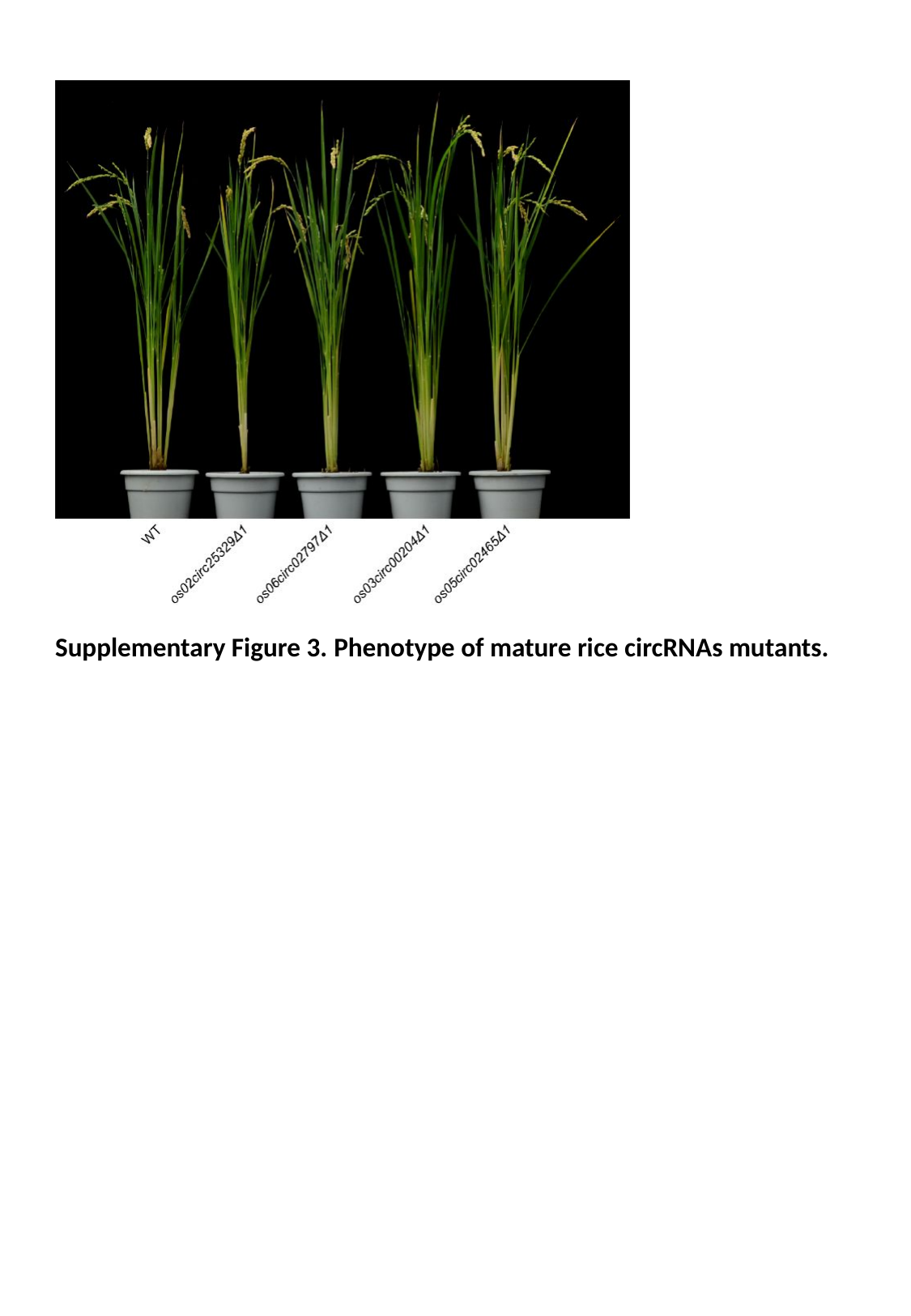

Supplementary Figure 3. Phenotype of mature rice circRNAs mutants.

## Slide 4
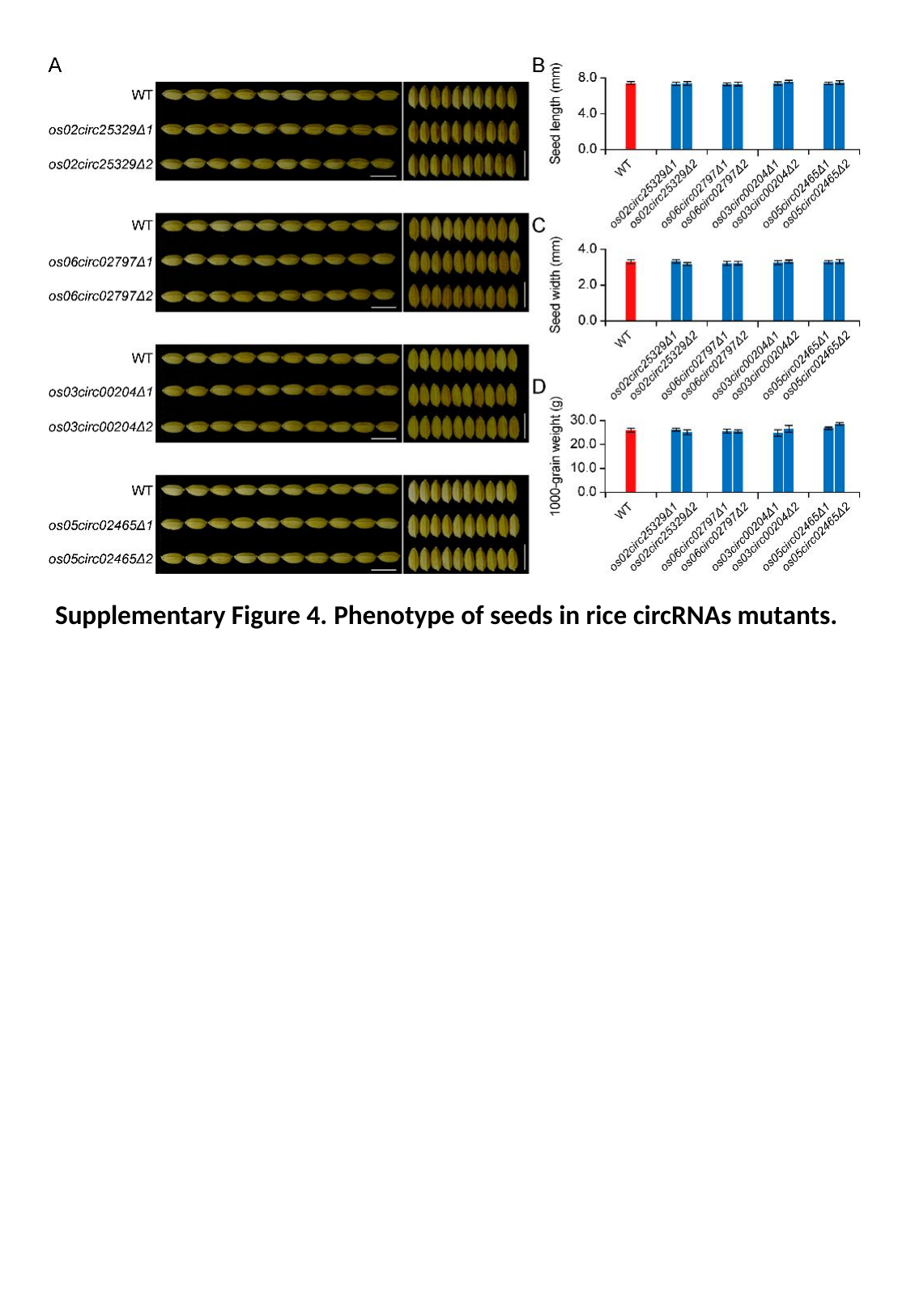

Supplementary Figure 4. Phenotype of seeds in rice circRNAs mutants.

## Slide 5
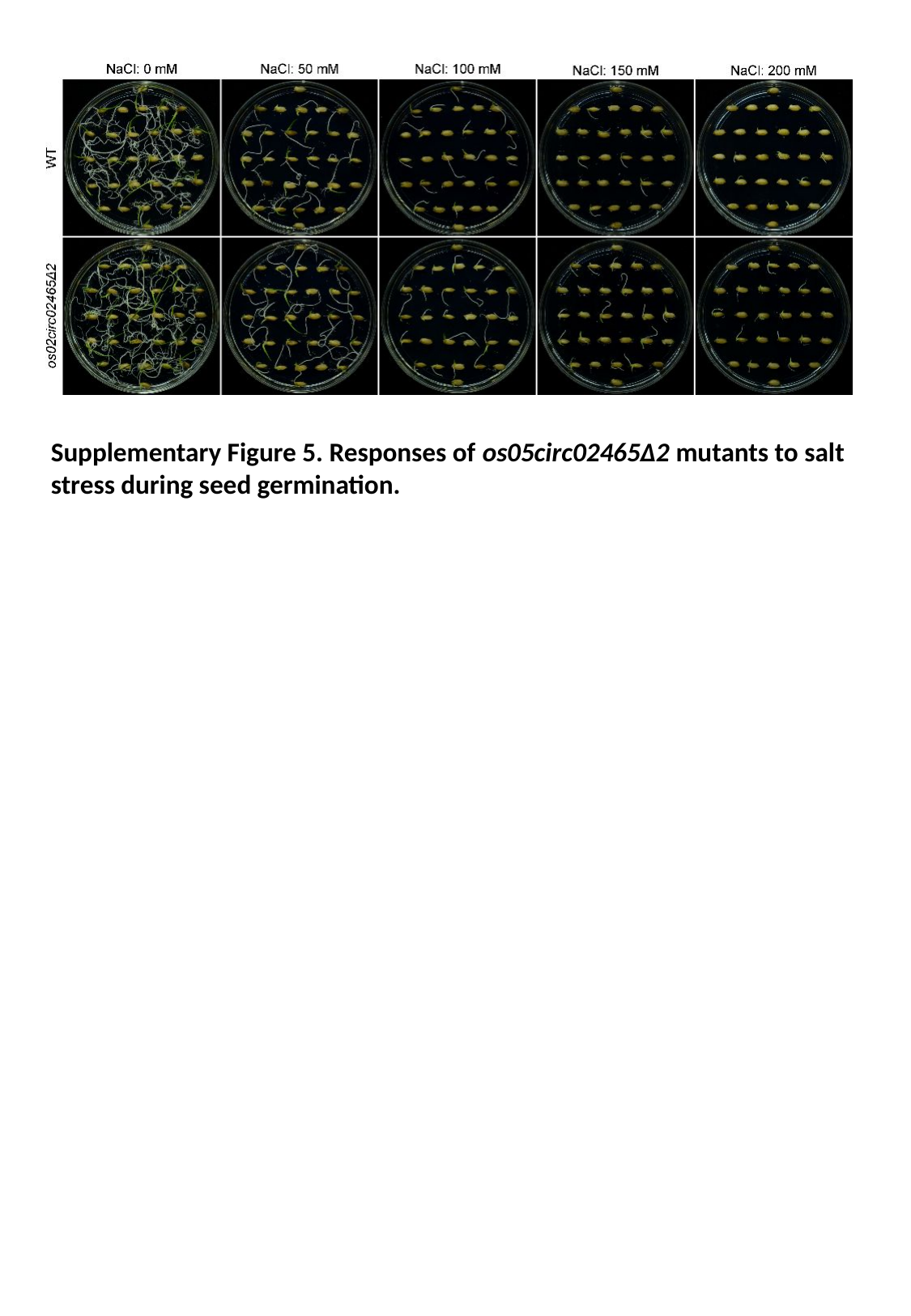

Supplementary Figure 5. Responses of os05circ02465∆2 mutants to salt stress during seed germination.

## Slide 6
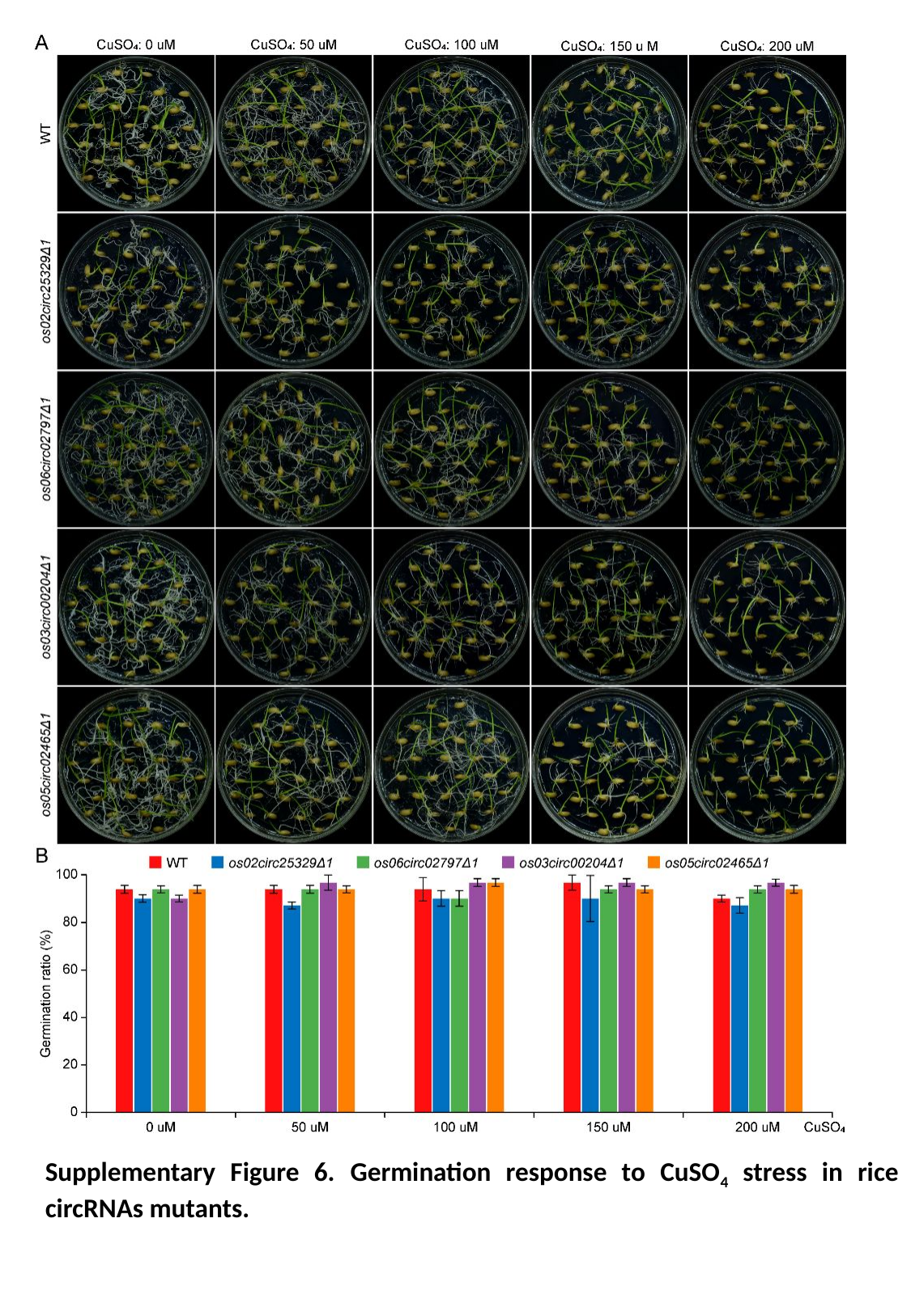

Supplementary Figure 6. Germination response to CuSO4 stress in rice circRNAs mutants.

## Slide 7
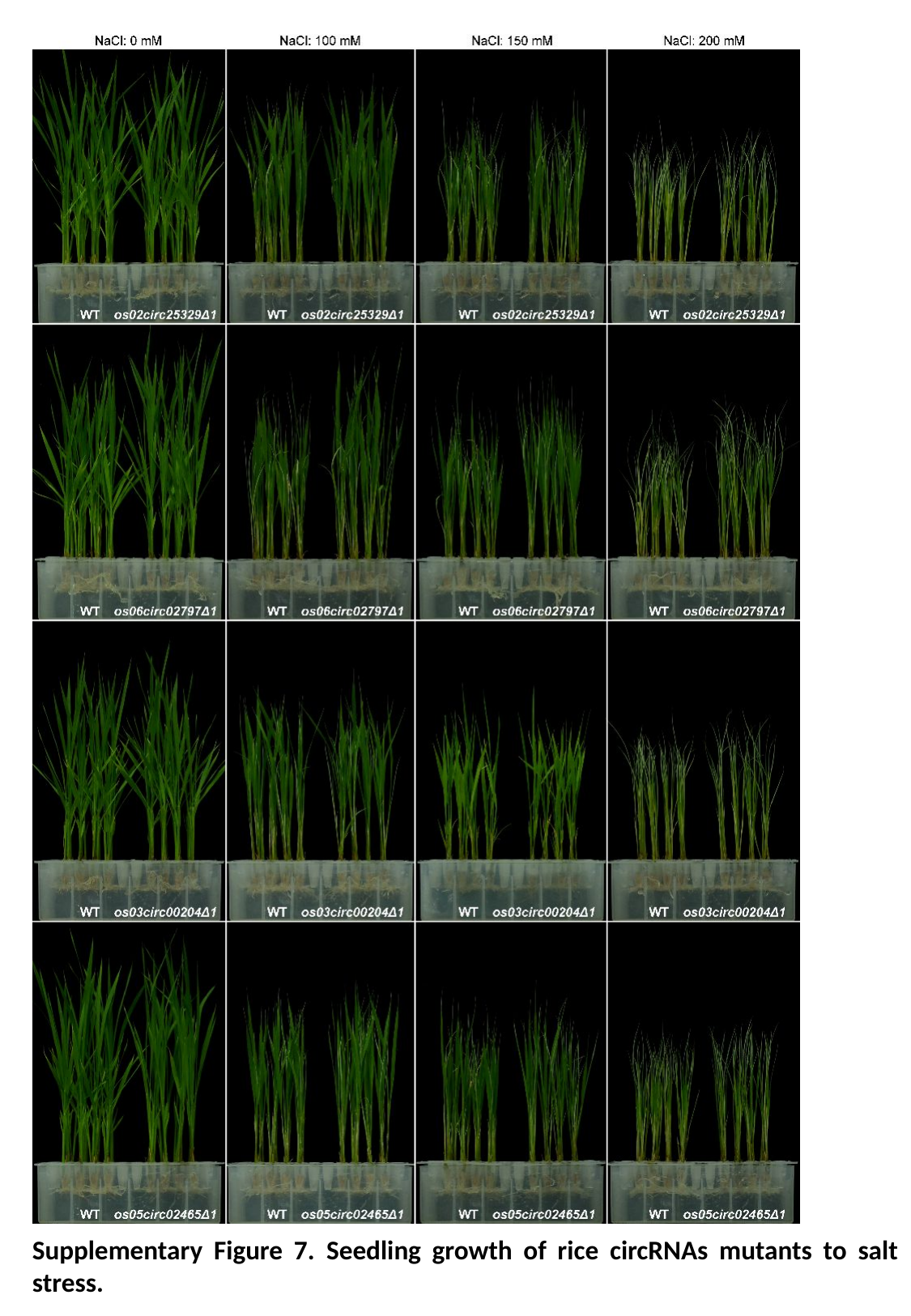

Supplementary Figure 7. Seedling growth of rice circRNAs mutants to salt stress.

## Slide 8
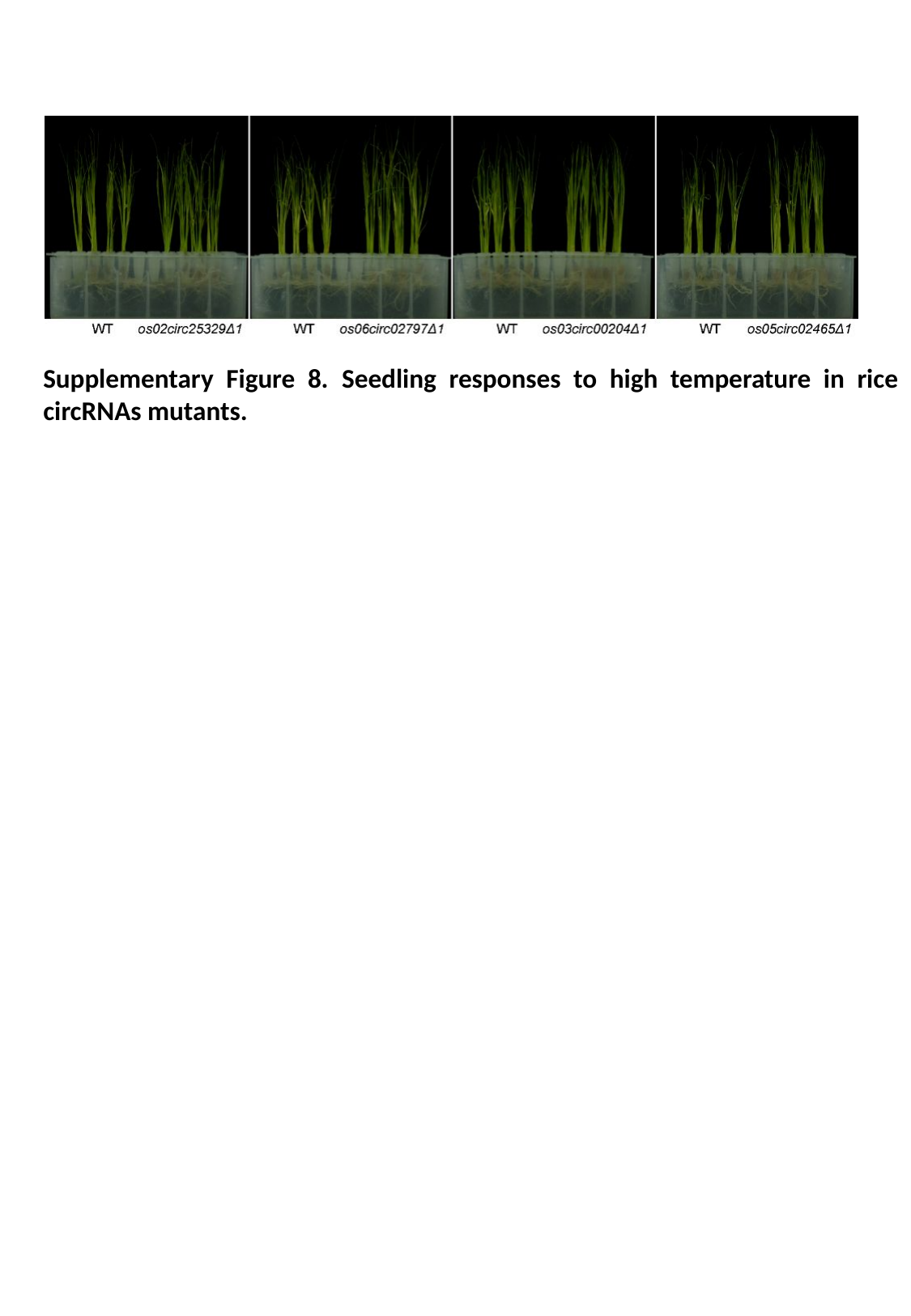

Supplementary Figure 8. Seedling responses to high temperature in rice circRNAs mutants.

## Slide 9
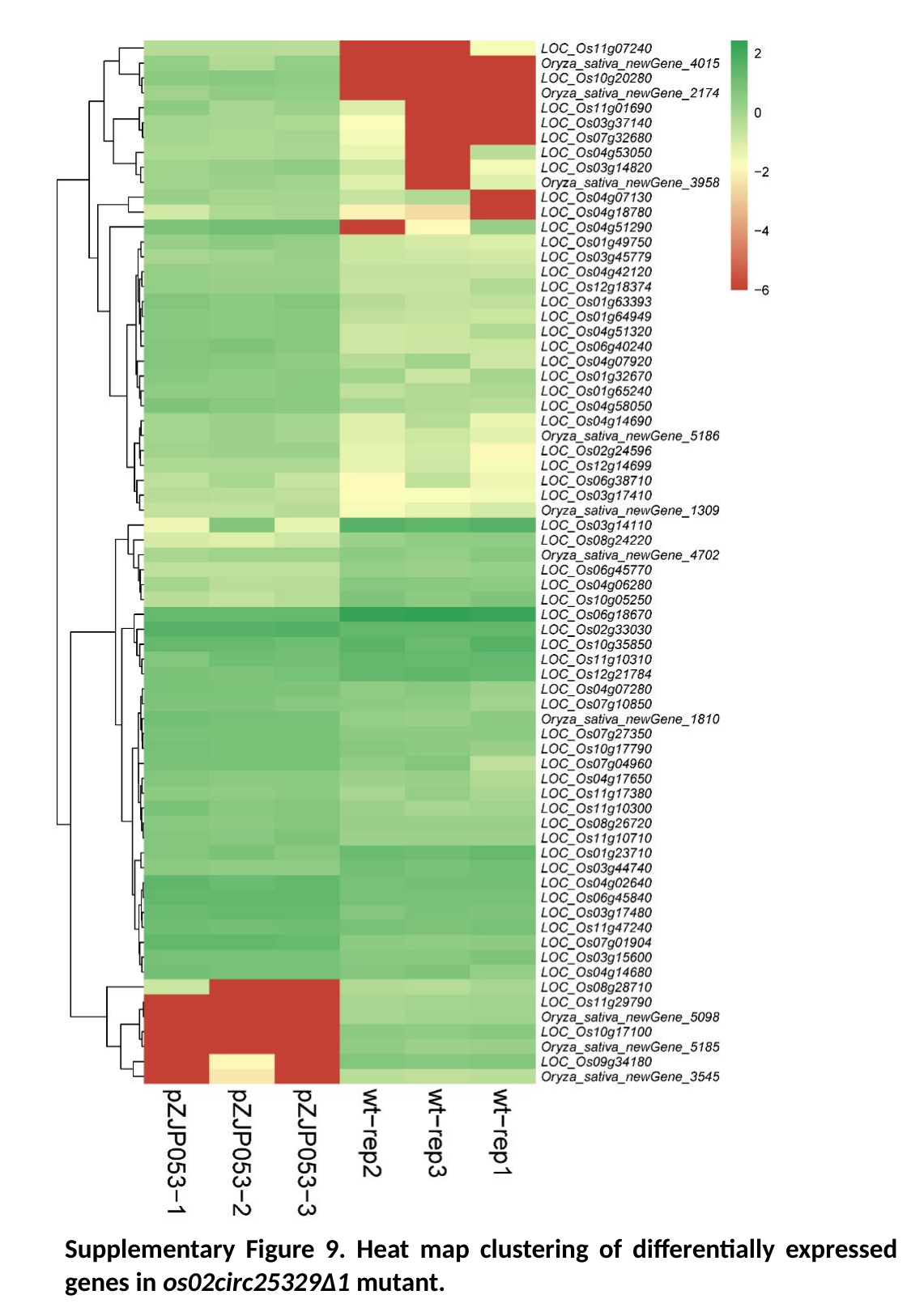

Supplementary Figure 9. Heat map clustering of differentially expressed genes in os02circ25329Δ1 mutant.

## Slide 10
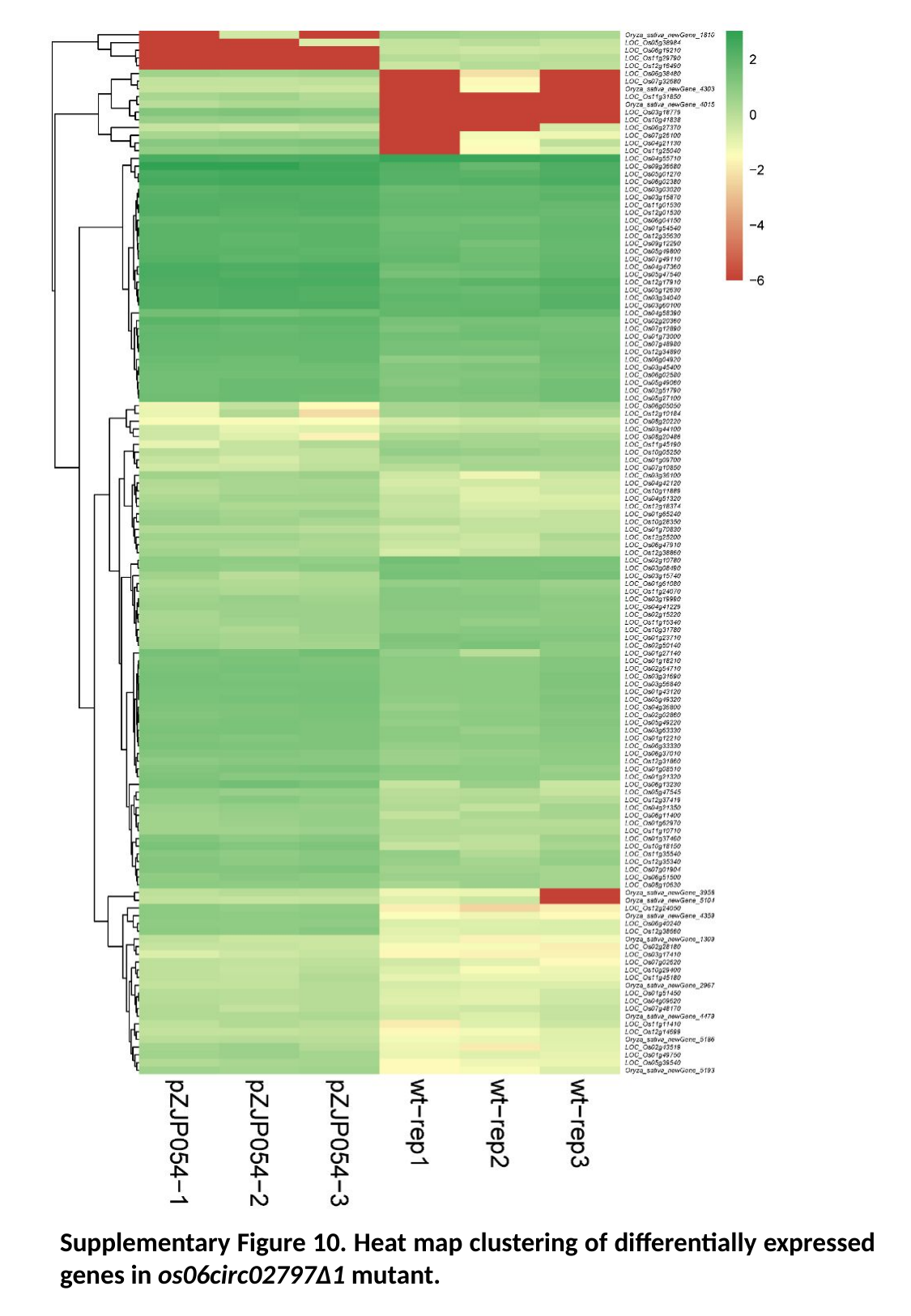

Supplementary Figure 10. Heat map clustering of differentially expressed genes in os06circ02797Δ1 mutant.

## Slide 11
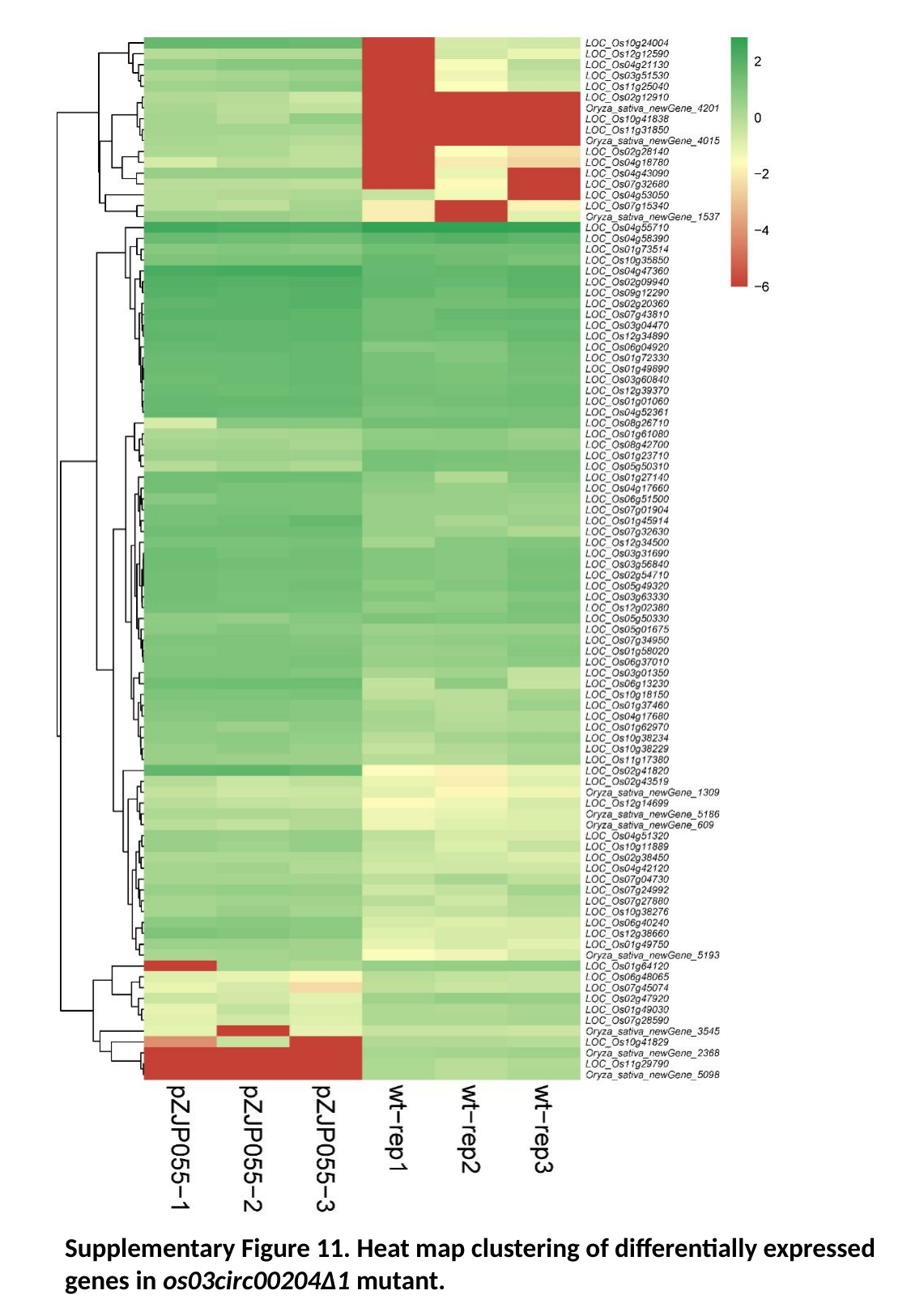

Supplementary Figure 11. Heat map clustering of differentially expressed genes in os03circ00204Δ1 mutant.

## Slide 12
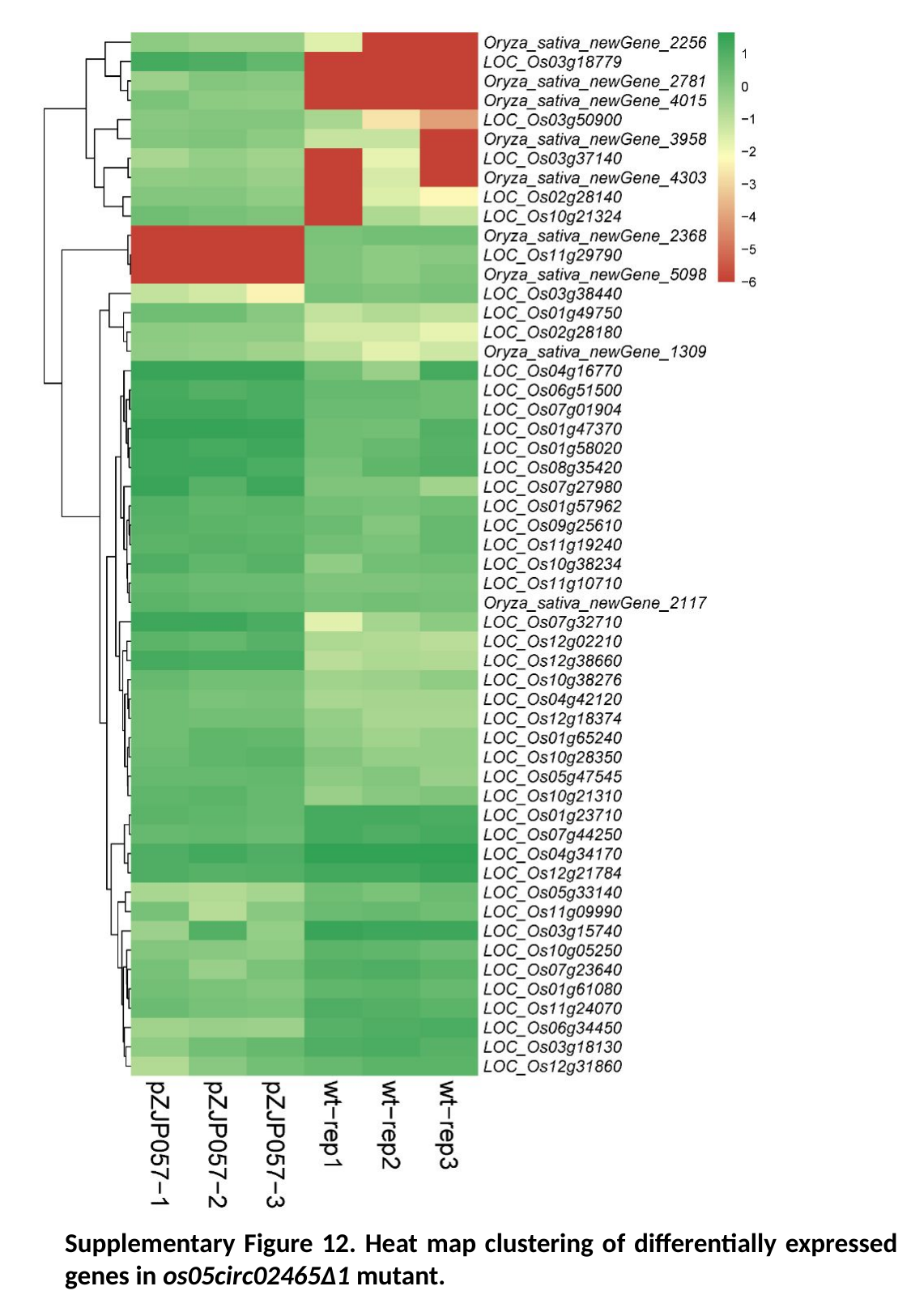

Supplementary Figure 12. Heat map clustering of differentially expressed genes in os05circ02465Δ1 mutant.

## Slide 13
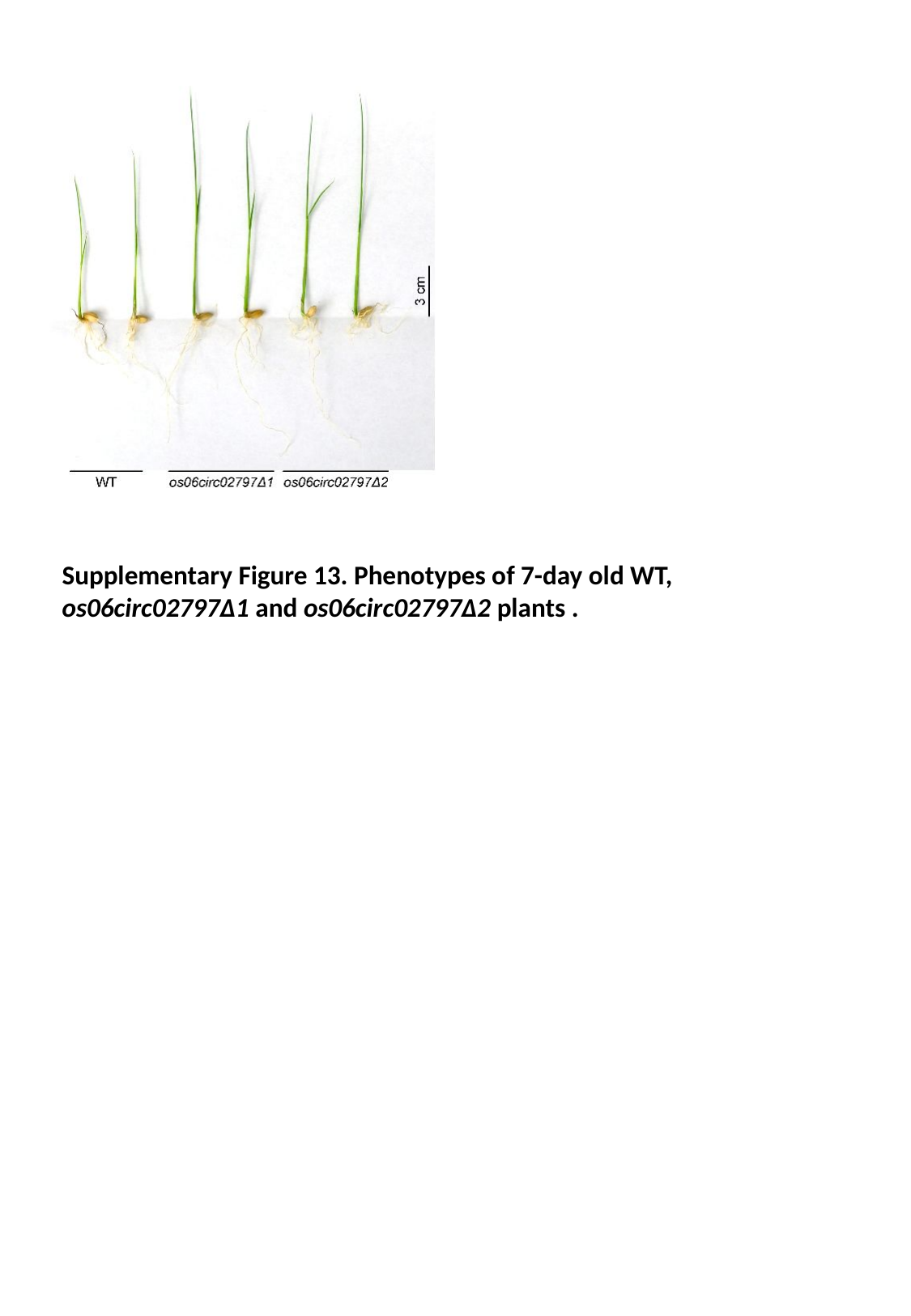

Supplementary Figure 13. Phenotypes of 7-day old WT, os06circ02797∆1 and os06circ02797∆2 plants .

## Slide 14
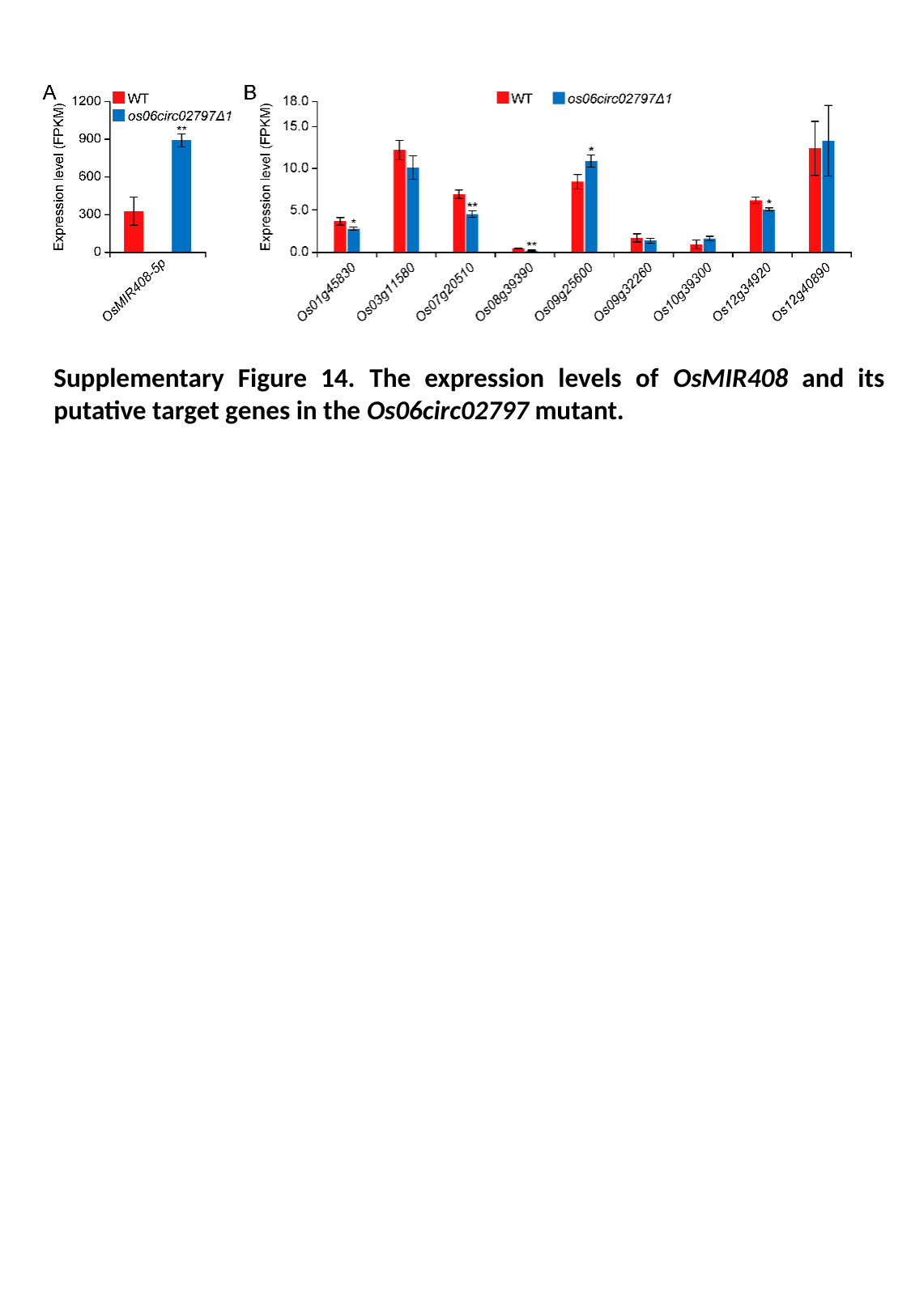

Supplementary Figure 14. The expression levels of OsMIR408 and its putative target genes in the Os06circ02797 mutant.
